# Attitudes toward driving after cannabis use: a systematic review

**DOI:** 10.1186/s42238-024-00240-0

**Published:** 2024-09-28

**Authors:** Bianca Boicu, Durr Al-Hakim, Yue Yuan, Jeffrey Brubacher

**Affiliations:** 1 Department of Emergency Medicine, University of British Columbia, Vancouver, BC Canada; 2 Vancouver, Canada

**Keywords:** Cannabis, Marijuana, Impaired driving, Attitudes, Beliefs, Risk perceptions

## Abstract

**Background:**

Driving after cannabis use (DACU) is associated with increased risk of motor vehicle collisions. As cannabis legalization expands, DACU is emerging as a major public safety concern. Attitudes have a significant impact on behavioural decision making. As such, understanding the degree to which people have favorable or unfavorable evaluations of DACU is an important first step for informing prevention efforts. This systematic review summarizes existing evidence on attitudes toward DACU, their association with actual or intended DACU, and changes in attitudes following legalization of recreational cannabis.

**Methods:**

Four electronic databases (MEDLINE, EMBASE, PsycINFO, and TRID) were searched for studies that reported attitudes or changes in attitudes toward DACU published between their inception dates and February 26 2024. A total of 1,099 records were retrieved. Studies were analyzed using an inductive thematic synthesis approach.

**Results:**

Seventy studies from seven countries originating predominantly from the United States and Canada met inclusion criteria. Thematic analysis identified six themes. (I) Attitudes toward the safety and acceptability of DACU are mixed; participants in 35 studies predominantly expressed negative attitudes toward DACU (e.g., DACU is dangerous, affects driving ability, and increases crash risk). However, 20 studies reported opposing views. (II) Attitudes toward DACU vary by age, sex/gender, and cannabis use frequency; youth, men, and frequent cannabis users tended to view DACU more favorably than older participants, women, and occasional or non-users. (III) Attitudes toward DACU are associated with past DACU and intention to DACU. (IV) DACU is viewed more favorably than driving after drinking alcohol. (V) The relationship between legal status of recreational cannabis and attitudes toward DACU is unclear. (VI) Perceived risk of apprehension for DACU is low to moderate.

**Conclusions:**

This review found that perceptions of DACU are primarily negative but mixed. Findings suggest that attitudes toward DACU are important targets for interventions to reduce this behaviour.

**Supplementary Information:**

The online version contains supplementary material available at 10.1186/s42238-024-00240-0.

## Introduction

As legalization of recreational cannabis expands worldwide, driving after using cannabis (DACU) becomes an increasingly relevant public safety concern. The prevalence of DACU has been increasing in Canada (Brubacher et al. 2022) and the United States (US), (Fink et al. 2020) with annual prevalence estimates from roadside surveys ranging from 4–10% in 2012–2022 (Beasley and Beirness 2012; Beirness 2018; Beirness 2022; Beirness et al. 2017; Johnson et al. 2012). Cannabis is frequently implicated in serious and fatal motor-vehicle collisions (MVCs), with odds ratios for collision risk ranging from 1.36 (95% CI: 1.15–1.61) (Rogeberg and Elvik 2016) to 1.92 (95% CI: 1.35–2.73), (Asbridge et al. 2012) although differences in study design make it difficult to ascertain the exact prevalence of MVCs attributable to cannabis. A growing body of epidemiological and experimental research shows that cannabis use leads to decrements in driving performance (Rogeberg and Elvik 2016; Asbridge et al. 2012; Li et al. 2012; Li et al. 2017; Hartman and Huestis 2013). In driver simulator and on-road studies, tetrahydrocannabinol (THC) impairs driving skills including lateral control, steering control, speed maintenance, and reaction time in both occasional and heavy users (Hartman and Huestis 2013; Arkell et al. 2020a; Bosker et al. 2012; Downey et al. 2013; Hartman et al. 2015; McCartney et al. 2021; Micallef et al. 2018; Ramaekers et al. 2000; Ronen et al. 2008; Brands et al. 2019).

To reduce the prevalence of DACU, it is necessary to evaluate factors that predict decisions to engage in this behaviour. If these factors can be modified, it may be possible to develop prevention programs to reduce the incidence of cannabis-related MVCs. Drug-driving prevention research has largely focused on cognitive predictors of intention to drive impaired, such as perceived risk (Earle et al. 2020; Ward et al. 2018; Ward et al. 2017; McCarthy et al. 2007; Malhotra et al. 2017). Broadly, prevention strategies aim to change traffic safety culture by increasing perceptions of dangerousness and severity of associated consequences (Shults et al. 2001). These efforts are based on behavioural theories such as the Theory of Reasoned Action, (Fishbein and Ajzen 1975) which explain and predict voluntary behaviour as a function of attitudes, subjective norms, and intentions. Specifically, attitudes (i.e., beliefs about a behaviour and evaluations of behavioural outcomes) and subjective norms (i.e., beliefs about the extent to which others encourage performance of a behaviour) jointly determine intentional performance of a behaviour. The Theory of Planned Behaviour (Ajzen 1991) adds control beliefs (i.e., perceived ability to perform or avoid a behaviour) as an antecedent of behavioural intention. These theories figure prominently in health promotion strategies (Godin and Kok 1996; Hausenblas et al. 1997; Hackman and Knowlden 2014). Following this line of reasoning, positive attitudes toward DACU play an important role in motivating intentions to engage in DACU. Indeed, safety perceptions have been shown to predict actual DACU (Aston et al. 2016; Arterberry et al. 2017; Borodovsky et al. 2020). Concerningly, existing cross-sectional research has shown that a large proportion of people believe they can safely DACU, and that some even report that cannabis makes them better drivers (Greene 2018; Swift et al. 2010).

Identifying individual factors associated with intention to DACU can further inform prevention strategies. For example, campaigns might be directed toward frequent cannabis users, who tend to report greater willingness and intention to DACU (Allen et al. 2016; Otto et al. 2016) relative to occasional and non-users. Situational factors can also predict DACU and provide interventional opportunities. For instance, some evidence suggests that the prevalence of moderately injured drivers with a blood THC level of 2ng/ml or more increased after legalization of recreational cannabis and introduction of cannabis retail markets, (Brubacher et al. 2022) while other evidence from drug use surveys suggests DACU has not increased (Government of Canada 2018).

Despite the growing body of research on this topic, a systematic review of attitudes toward DACU has not been conducted, to our knowledge. This review aims to characterize attitudes toward DACU. Attitudes are the degree to which people have positive or negative evaluations of a behaviour (e.g., DACU is dangerous or unacceptable) and entail consideration of the expected consequences (e.g., DACU is likely to lead to an MVC). This systematic review aims to provide a broad, comprehensive overview of quantitative, qualitative, and mixed-methods research related to DACU attitudes using an inductive thematic synthesis approach. Thematic analysis is useful for identifying, interpreting, and synthesizing key features across various types of data, allowing for a nuanced account of DACU attitudes.

## Methods

### Search strategy and data sources

This systematic review was guided by the Preferred Reporting Items for Systematic Reviews and Meta-Analysis (PRISMA) Statement (Page et al. 2021). Studies were identified by searching four electronic databases: MEDLINE (Ovid), EMBASE (Ovid), PsycINFO (EBSCOhost), and Transport Research International Documentation. All databases were initially searched from their inception dates until May 31 2022 and the search was later updated to include studies published up to February 26, 2024. Key terms relating to cannabis use (e.g., cannabis OR marijuana OR THC), attitudes (e.g., attitude* OR belie* OR opinion*), and driving (e.g., driv* OR motor vehicle OR DACU) were combined. The keyword search terms were adapted for each database (see Additional File 1). All articles were uploaded to Covidence, (Covidence 2023) an online platform that streamlines the production of systematic reviews and allows for asynchronous collaboration among reviewers. Two reviewers (B.B. and Y.Y.) independently screened all studies by title and abstract. Full text of the retained studies was independently reviewed by two reviewers (B.B. and D.A.). Disagreements in both stages were resolved by consensus or involving a fourth reviewer (J.B.). The protocol was pre-registered on PROSPERO (CRD42022337260).

### Eligibility criteria

Studies that met the following criteria were included: (i) report attitudes or changes in attitudes toward cannabis use and driving, (ii) report qualitative, quantitative, or mixed methods empirical research, (iii) use primary research data, (iv) English-language, and (v) full-text available. Studies reporting secondary research data were excluded but used to identify the original study. Studies that met the following criteria were excluded: (i) attitudes or changes in attitudes toward cannabis use and driving not reported, (ii) narrative non-research reports (e.g., commentaries, position statements), (iii) not in English, (iv) full text not available. Both academic and gray literature (e.g., reports, policy literature) were included. As this review aimed to synthesize all available data on attitudes toward DACU, no studies were excluded based on study design or sample characteristics. Where two or more studies used the same data-set, the report with the largest sample size was retained.

### Data extraction and quality assessment

Data from eligible studies were collected in a pre-developed data extraction form by two reviewers (B.B. and D.A.) using Microsoft Excel. Extracted data included: (i) title, (ii) author(s), (iii) year of publication, (iv) location from which subjects were sampled, (v) aim(s) and hypothesis(es), (vi) sample characteristics, including sample size, age, sex/gender, cannabis use history, and inclusion/exclusion criteria, (vii) data collection date(s), (viii) study design/data collection method(s), (ix) outcome measures, (x) data analysis method(s), (xi) control for confounding variables, and (xii) key findings. Both reviewers performed extraction for 10 studies and cross-checked the extracted data to assess agreement. Each reviewer then extracted data for approximately half of the remaining 60 studies.

Methodological quality and risk of bias was assessed by two reviewers (B.B. and D.A.) using the Standard Quality Assessment Criteria for Evaluating Primary Research Papers (SQAC) (Kmet et al. 2004). The SQAC comprises separate checklists for quantitative and qualitative studies; both checklists were used to assess mixed-method studies. A conservative inclusion threshold of 75% was used (Kmet et al. 2004) (no studies were excluded on this basis). Additional File 2 provides a summary of the included studies.

### Data synthesis

An inductive thematic synthesis approach (Ryan et al. 2018) was used to identify, analyze, and report patterns across findings from included studies. First, two reviewers (B.B. and D.A.) read the original texts, adding to a bank of free codes as necessary. Decisions to combine, rename, or eliminate these initial codes were reached through discussion. This process yielded 48 initial codes (see Additional File 3). Each code was entered as a row in a table and populated with data from relevant studies. Studies reporting any finding related to the code were added to the table, including those reporting non-significant results. Reviewers then grouped initial codes into a hierarchical structure by identifying similarities between the codes. Analytic themes were generated by synthesizing extracted findings in relation to the research question. Both reviewers reviewed themes and assigned theme names.

## Results

### Description of included studies

**Fig. 1:**
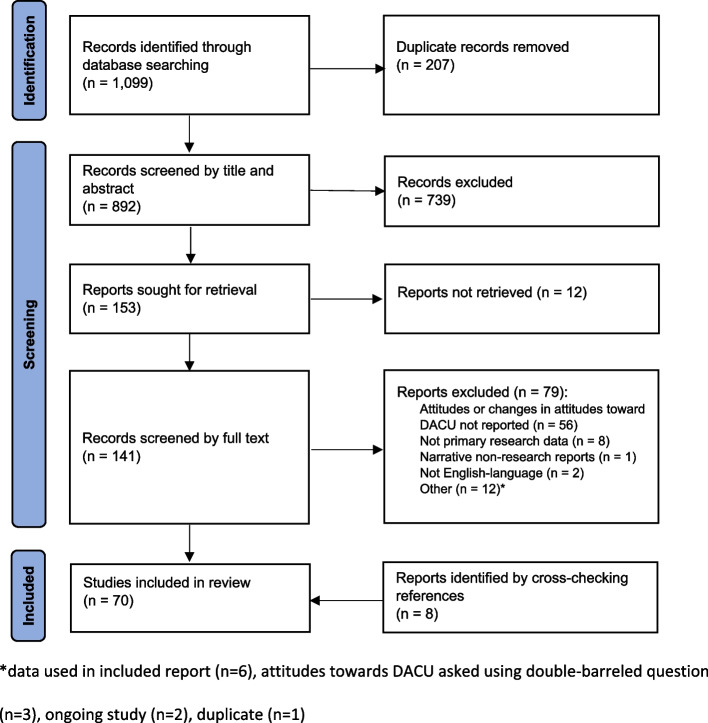
PRISMA screening process with reasons for exclusion

A total of 1,099 non-duplicate records were screened by title and abstract, resulting in 141 records retrieved for full text review. Seventy-nine records were excluded for reasons shown in Fig. 1 and eight records were added by cross-checking references from the 62 included studies. Data were extracted from a final set of 70 studies. Inter-rater reliability was substantial for the abstract and title screening (kappa = 0.60, 0.59) and full text review (kappa = 0.62, 0.79) during the initial and updated searches, respectively. (McHugh 2012).

The 70 included articles dated from 1981 to 2024, with only three studies published before 2000. The synthesis of findings involved over 126,930 participants. Twenty-seven studies sampled youth, young adults (< 35 years old) or students. Twenty-four samples were restricted to people who had used cannabis (including two studies with medical cannabis users). Twenty-six studies were restricted to participants who drove (at least once in the past year) or held a driving license. Five studies drew from samples of drug users (*n* = 3), police detainees (*n* = 1), or participants in a remedial program for impaired drivers (*n* = 1).

Male and female subjects were equally represented (49% male), although seven studies did not provide sex or gender demographics. Most studies sampled subjects from the US (37 studies) or Canada (17 studies); the remaining studies included subjects from Australia (*n* = 11), the United Kingdom (*n* = 4), New Zealand (*n* = 2), Spain (*n* = 2), and Israel (*n* = 1). This count totals more than 70 because three studies used international samples. Data was collected primarily through online, in-person, and telephone surveys or questions (*n* = 54), in-person or telephone interviews (*n* = 15), and focus groups (*n* = 6). These counts total more than 70 because five studies used multiple data collection formats. Three pairs of studies were suspected of having fully (Allen et al. 2016; Davis et al. 2016) or partially (Wadsworth and Hammond 2018; Wadsworth and Hammond 2019; Mills et al. 2023; Mills and Freeman 2023) overlapping sample frames; all were retained because they reported different analyses. Table 1 summarizes the included studies.

**Table 1:** Characteristics of included studies

Characteristic	Number of studies
Study population	
Youth/young adults/students	27
Cannabis users	24
Drivers	26
Special population	5
Country	
US	37
Canada	17
Australia	11
United Kingdom	4
New Zealand	2
Spain	2
Israel	1
Data collection method	
Surveys	54
Interview	15
Focus group	6

### Study findings

Analysis of the studies identified six themes (Table 2).

**Table 2:** Summary of themes

Theme	Summary
Attitudes toward the safety and acceptability DACU are mixed	Sixty studies (*n* > 110,289 participants; NR in one study) supported this theme. Participants in 35 studies (*n* = 67,761), predominantly expressed negative attitudes toward DACU. Participants in 20 studies (*n* > 23,077, NR in one study) expressed the opposite. Participants in 5 studies (*n* = 19,451) expressed neutral or mixed views. Subsets of participants in 16 studies (*n* > 8,039, NR in one study) reported that cannabis can improve or have positive effects on driving ability.
Attitudes toward DACU can differ by age, sex/gender, and cannabis use frequency	Age. Nineteen studies (*n* = 40,006) supported this theme. Youth were more likely to hold positive attitudes toward DACU than older participants across ten studies (*n* = 30,603). One study found the reverse (*n* = 1,773) and eight studies found that older age was unrelated to attitudes (*n* = 7,630). Sex/gender. Twenty-six studies (*n* = 46,694) supported this theme. Men were more likely to hold positive attitudes toward DACU than women across 13 studies (*n* = 33,376). Two studies reported the reverse (*n* = 3,416), two reported mixed results (*n* = 2,842), and nine found no differences by gender (*n* = 7,060). Cannabis use frequency. Twenty-four studies (*n* = 26,637) supported this theme. Frequent users had more positive attitudes toward DACU, reported greater intention to DACU, more frequent past DACU, and lower likelihood of legal consequences than those who used cannabis less frequently across 23 studies (*n* = 26,378). One study reported no such relationship (*n* = 259).
Attitudes toward DACU are associated with past DACU and intention to DACU	Twenty-six studies (*n* = 28,775) supported this theme. Positive attitudes toward DACU were associated with actual DACU and greater intention and willingness to DACU.
DACU is viewed more favorably than driving after drinking alcohol	Thirty-seven studies (*n* > 56,159, NR in one study) supported this theme. Attitudes toward DACU were more positive than attitudes toward driving after drinking alcohol in 31 studies (*n* > 49,420, NR in one study). Five studies reported contradictory results (*n* = 6,672). There was consensus that driving under the combined influence of cannabis and alcohol was riskier than DACU alone across six studies (*n* = 1,305).
The relationship between legal status of recreational cannabis and attitudes toward DACU is unclear	Fifteen studies (*n* = 56,127) supported this theme. Opinions toward the DACU safety and related laws differed across five studies (*n* = 37,477). Five studies (*n* = 12,566) did not identify any clear differences. Five studies (*n* = 6,084) reported various effects of legalization (e.g., anticipated post-legalization rise in DACU prevalence).
Perceived risk of apprehension for DACU is low to moderate	Thirty-one studies (*n* = 69,503) supported this theme. Perceived risk of apprehension and/or penalty for DACU was low to moderate, especially relative to driving after alcohol, in 25 studies (*n* = 54,433). One study had contradictory results (*n* = 416). Fourteen studies discussed the effect of and support for preventive policies, penalties, and detection efforts (*n* = 26,941).

### Attitudes toward the safety and acceptability DACU are mixed

Sixty studies from the US (*n* = 29, 1981–2024), Canada (*n* = 13, 2006–2023), Australia (*n* = 11, 2000–2023), England (*n* = 2, 2003–2005), New Zealand (*n* = 2, 2009–2017), Spain (*n* = 2, 2016–2022), and Canada, the US, and England (*n* = 1, 2019) reported beliefs about the effect of cannabis on driving safety, driving ability, and crash risk. In 35 studies, participants predominantly expressed negative attitudes toward DACU or did not endorse statements such as ‘DACU is safe’. Specifically, DACU was considered to be a dangerous, unsafe, or risky behavior that increases collision risk and cannabis was thought to negatively affect driving ability. DACU was also considered to be unacceptable and unenjoyable (Jones et al. 2006; Earle et al. 2020; Ward et al. 2018; Ward et al. 2017; Malhotra et al. 2017; Arterberry et al. 2017; Swift et al. 2010; Allen et al. 2016; Otto et al. 2016; Davis et al. 2016; EKOS Research Associates Inc. 2017; Corporate Research Associates 2018; Benedetti et al. 2021; AAA Foundation for Traffic Safety 2018; Cavazos-Rehg et al. 2018; Duckworth and Lee 2019; Eichelberger 2016; Fischer et al. 2006; Ginsburg et al. 2008; Goodman et al. 2020; Grilly 1981; Hammond 2009; Jonah 2013; Kohn et al. 2014; Lenné et al. 2001; Lensch et al. 2020; McDonald et al. 2021; McKiernan and Fleming 2017; Pino et al. 2016; Porath-Waller 2008; Terry and Wright 2005; Wechsler et al. 1984; Eichelberger 2023; Ortiz-Peregrina et al. 2022; Wickens et al. 2023). These studies were largely from the US (*n* = 20) and Canada (*n* = 9), and eight were from Australia (*n* = 3), Spain (*n* = 2), New Zealand (*n* = 2), and England (*n* = 1). Conversely, participants predominantly reported positive attitudes toward DACU in 18 studies. Namely, participants expressed that DACU is safe, does not increase collision risk, does not impair driving ability, or is acceptable (Fischer et al. 2014; Aston et al. 2016; Greene 2018; Mills et al. 2023; Mills and Freeman 2023; Adams et al. 2008; Aitken et al. 2000; Arterberry et al. 2013; Berg et al. 2018; Colonna et al. 2021; Cuttler et al. 2018; Danton et al. 2003; Huỳnh et al. 2022; Teeters et al. 2021; Townsend et al. 1996; Barrie et al. 2011; Hultgren et al. 2023a; Miller et al. 2024). Two studies found that participants who used cannabis for therapeutic reasons did not think it impaired their driving (Arkell et al. 2020b; Arkell et al. 2023a). These studies were largely from the US (*n* = 9), Australia (*n* = 7) and four were from Canada (*n* = 3) and England (*n* = 1). Participants in five studies expressed neutral or mixed views (Wadsworth and Hammond 2018; Wadsworth and Hammond 2019; Eichelberger 2019; Matthews et al. 2014; Wickens et al. 2019). Interestingly, negative attitudes toward DACU were more common among studies conducted with non-medical users residing in areas with legalized recreational cannabis. This observation is supported by a US study that found that the belief that DACU is unsafe is more common in states with legalized sale of recreational cannabis (aPR = 1.10), (Lensch et al. 2020) although evidence is mixed (see theme V).

Across studies that asked about the impact of DACU on driving, subsets of participants in nine studies identified driving-relevant skills that may be negatively impacted by cannabis use. These included slowed reaction time, impaired attention or concentration, distorted visual perception, and paranoia (EKOS Research Associates Inc. 2017; Lenné et al. 2001; McKiernan and Fleming 2017; Terry and Wright 2005; Aitken et al. 2000; Colonna et al. 2021; Arkell et al. 2023a; Wickens et al. 2019; MacDonald et al. 2008). Such beliefs may be protective, as subjects who avoided or decided against DACU cited safety concern as a primary reason (Malhotra et al. 2017; Swift et al. 2010; Hammond 2009). However, subsets of participants in 16 of these studies reported that using cannabis actually improves or has positive effects on drivers (Greene 2018; Swift et al. 2010; Otto et al. 2016; EKOS Research Associates Inc. 2017; Cavazos-Rehg et al. 2018; Lenné et al. 2001; McKiernan and Fleming 2017; Terry and Wright 2005; Adams et al. 2008; Colonna et al. 2021; Danton et al. 2003; Barrie et al. 2011; Miller et al. 2024; Arkell et al. 2020b; Wickens et al. 2019; MacDonald et al. 2008). This belief may be attributed to the notion that cannabis makes drivers more cautious, relaxed, alert, or calm, or makes them drive slowly (Swift et al. 2010; Otto et al. 2016; EKOS Research Associates Inc. 2017; McKiernan and Fleming 2017; Terry and Wright 2005; Danton et al. 2003; Arkell et al. 2020b; Wickens et al. 2019; MacDonald et al. 2008; Barrie et al. 2011). Indeed, Otto et al. found that those who had a positive attitude toward DACU were more likely to believe that they would be more alert, calm, and cautious if they drove after using cannabis (Otto et al. 2016). The amount of cannabis consumed, tolerance, frequency of use, and time waited before driving were thought to moderate the effect of cannabis on driving ability (Greene 2018; McKiernan and Fleming 2017; Porath-Waller 2008; Danton et al. 2003; Wickens et al. 2019). Five studies identified gaps between personal and others’ attitudes toward DACU; participants were more likely to indicate that they were less impaired when DACU and less likely to have an accident, intend to drive, or hold positive attitudes toward DACU than others (Ward et al. 2017; Swift et al. 2010; Fischer et al. 2006; Grilly 1981; Wickens et al. 2019).

### Attitudes toward DACU can differ by age, sex/gender, and cannabis use frequency

#### Age

Nineteen studies from the US (*n* = 12, 2016–2023), Canada (*n* = 6, 2013–2023), and Australia (*n* = 1, 2020) reported differences in attitudes toward DACU by age. Younger participants were more likely than older participants to perceive DACU as an acceptable, safe, or unconcerning behavior that does not negatively affect driving ability or increase MVC risk (EKOS Research Associates Inc. 2017; Corporate Research Associates 2018; Benedetti et al. 2021; AAA Foundation for Traffic Safety 2018; Eichelberger 2016; Jonah 2013; Eichelberger 2023). Youth also reported greater willingness and intention to DACU than older participants (Ward et al. 2018; Davis et al. 2016). One US study found that older participants rated medical, but not recreational, cannabis use as having a more negative impact on driving ability (Auguste and Zambrano 2023). Eight studies found that older age was unrelated to attitudes toward DACU after use of cannabis (Aston et al. 2016; Allen et al. 2016; Duckworth and Lee 2019; Goodman et al. 2020; McDonald et al. 2021; Wickens et al. 2023; Arkell et al. 2020b; Eichelberger 2019) and one reported that older age was associated with believing that DACU is safe (Cuttler et al. 2018).

It is important to note that safety beliefs varied among other studies studies conducted with samples of youth. While teenagers and youth predominantly report that DACU is dangerous and cannabis negatively affects driving ability, some youth report that cannabis use could actually improve driving ability (Greene 2018; EKOS Research Associates Inc. 2017; Lenné et al. 2001; Porath-Waller 2008; Danton et al. 2003). As in drivers of all ages, youth believed effects depended on the amount of cannabis consumed, tolerance, and time waited before driving (Greene 2018; McKiernan and Fleming 2017; Porath-Waller 2008; Danton et al. 2003).

#### Sex and gender

‘Sex’ and ‘gender’ is presented as one concept because the reviewed literature used these terms interchangeably. Twenty-six studies from the US (*n* = 13, 2007–2023), Canada (*n* = 7, 2008–2023), Australia (*n* = 5, 2001–2023) and Spain (*n* = 1, 2016) reported sex and gender differences in attitudes toward DACU. Compared to women, men were more likely to report that DACU is acceptable and safe and less likely to agree DACU is impairing, risky, or concerning (Earle et al. 2020; McCarthy et al. 2007; Corporate Research Associates 2018; Benedetti et al. 2021; Duckworth and Lee 2019; Eichelberger 2016; Jonah 2013; McDonald et al. 2021; Pino et al. 2016; Porath-Waller 2008; Arterberry et al. 2013; Brown et al. 2022). Men also believed that DACU is less dangerous than driving after drinking alcohol, (EKOS Research Associates Inc. 2017) reported less support for per-se cannabis laws, (Benedetti et al. 2021) greater intention to DACU, (Earle et al. 2020) and were less likely to believe that drivers who DACU would be stopped or charged by police (Allen et al. 2016; Corporate Research Associates 2018; Jonah 2013). However, these findings were not consistent across all studies. Eight studies found no association between gender and perceived safety of DACU, cannabis-related crash risk or impact of cannabis on driving ability, (Aston et al. 2016; Swift et al. 2010; Allen et al. 2016; Goodman et al. 2020; Eichelberger 2023; Wickens et al. 2023; Arkell et al. 2020b; Auguste and Zambrano 2023) likelihood of future DACU, (Jones et al. 2006) or likelihood of being stopped or charged by police (Goodman et al. 2020). Two studies reported mixed results depending on attitudinal probes or time of survey (Mills et al. 2023; Eichelberger 2019). Further, three studies found that men were more likely than women to admit that cannabis use impairs driving ability, (Lenné et al. 2001) less likely to approve of DACU, (AAA Foundation for Traffic Safety 2018) and more aware that DACU and driving after alcohol use could have the same legal penalties (Corporate Research Associates 2018).

#### Cannabis use frequency

Twenty-four studies from the US (*n* = 12, 1981–2023), Canada (*n* = 8, 2006–2022), Australia (*n* = 2, 2010–2020), New Zealand (*n* = 1, 2017) and Spain (*n* = 1, 2016) considered differences in attitudes toward DACU by cannabis use frequency. Participants who used cannabis more frequently were less likely to believe that driving ability is impaired by cannabis, that higher intoxication levels are safe for DACU, that DACU is dangerous, unsafe, risky, unacceptable, concerning or problematic, (McCarthy et al. 2007; Malhotra et al. 2017; Arterberry et al. 2017; Borodovsky et al. 2020; Swift et al. 2010; Allen et al. 2016; Otto et al. 2016; EKOS Research Associates Inc. 2017; Goodman et al. 2020; Grilly 1981; McDonald et al. 2021; Pino et al. 2016; Wechsler et al. 1984; Eichelberger 2023; Arterberry et al. 2013; Berg et al. 2018; Colonna et al. 2021; Cuttler et al. 2018; Huỳnh et al. 2022; Arkell et al. 2020b; Wickens et al. 2019; Auguste and Zambrano 2023) than those who used cannabis less frequently (including non-users). Frequent users also reported greater willingness and intention to DACU in the future (Allen et al. 2016; Otto et al. 2016; Fischer et al. 2006) than less frequent users. Only one study found no association between cannabis use frequency and believing that DACU increases collision risk (Wickens et al. 2023).

### Attitudes toward DACU are associated with past DACU and intention to DACU

Twenty-six studies from the US (*n* = 16, 2007–2023), Canada (*n* = 5, 2008–2021), and Australia (*n* = 5, 2010–2023) examined associations between attitudes toward DACU and actual or intended DACU. In the US, participants with a history of DACU had more favorable attitudes toward DACU than those who reported never or less frequent DACU (Ward et al. 2018; McCarthy et al. 2007; Aston et al. 2016; Arterberry et al. 2017; Otto et al. 2016; Davis et al. 2016; Cavazos-Rehg et al. 2018; Eichelberger 2016; Arterberry et al. 2013; Berg et al. 2018; Cuttler et al. 2018; Scott et al. 2021). For example, positive attitudes were associated with reporting past DACU (RR = 1.59) (Ward et al. 2018) while negative attitudes were associated with lower odds of driving five or more times within one hour of using cannabis (OR = 0.26) (Davis et al. 2016). Those who had ever engaged in DACU also believed that DACU was more prevalent and acceptable (Ward et al. 2018; McCarthy et al. 2007; Otto et al. 2016; Hultgren et al. 2023a) than those who had not. Greater intoxication levels perceived as safe for DACU also corresponded to more frequent DACU; for each unit increase in intoxication level perceived as safe for driving (0; sober/need to be sober to 10; so high that you throw up/vomit), the odds of past-month DACU increased 18–68% (MOR = 1.18–1.68) (Borodovsky et al. 2020). Interestingly, a third (30%) of participants who perceived their driving to be impaired by cannabis also reported DACU (Cuttler et al. 2018). Positive attitudes toward DACU also predicted greater willingness and intention to DACU in the future, which were in turn associated with engagement in DACU (Earle et al. 2020; Ward et al. 2018; Ward et al. 2018; Ward et al. 2017; Otto et al. 2016; Otto et al. 2016; Scott et al. 2021).

Studies conducted in Canada reported similar findings; having a history of DACU was associated with more positive attitudes toward DACU (Fischer et al. 2014; Goodman et al. 2020; McDonald et al. 2021; Colonna et al. 2021). Positive attitudes toward DACU also predicted greater willingness and intention to DACU in the future (Porath-Waller 2008). Similarly, in five studies conducted in Australia, participants who reported past or intended DACU were more likely to believe cannabis does not impair driving ability (OR = 3.53) or increase crash risk, and believing that cannabis increases accident risk discourages frequent engagement in DACU (OR = 0.4) (Swift et al. 2010; Mills et al. 2023; Mills and Freeman 2023; Arkell et al. 2023a; Matthews et al. 2014). Overall, the available evidence is consistent across the three countries and recreational or medical legal status of cannabis. However, it is worth noting that one international study found that US youth were more likely than Canadian or English youth to report DACU but less likely to report that DACU is risky (Wadsworth and Hammond 2019).

### DACU is viewed more favorably than driving after drinking alcohol

Thirty-seven studies from Canada (*n* = 12, 2006–2023), the US (*n* = 14, 1981–2023), US and Israel (*n* = 1, 2024), Australia (*n* = 5 2000–2014), England/United Kingdom (UK) (*n* = 3, 2000–2005), and New Zealand (*n* = 2, 2009–2017), examined perceptions of DACU relative to driving after drinking alcohol (and in some cases illicit drugs such as heroin or ecstasy). DACU was considered to be relatively less dangerous, impairing, risky, or problematic across 31 studies from Canada, (Corporate Research Associates 2018; Fischer et al. 2006; Jonah 2013; McKiernan and Fleming 2017; Wickens et al. 2023; Colonna et al. 2021; Wickens et al. 2019; MacDonald et al. 2008) the US, (McCarthy et al. 2007; Greene 2018; Allen et al. 2016; Davis et al. 2016; AAA Foundation for Traffic Safety 2018; Cavazos-Rehg et al. 2018; Duckworth and Lee 2019; Eichelberger 2016; Ginsburg et al. 2008; Grilly 1981; Kohn et al. 2014; Lensch et al. 2020; Eichelberger 2023; Hultgren et al. 2023b) Australia, (Swift et al. 2010; Aitken et al. 2000; Barrie et al. 2011; Matthews et al. 2014) England/UK, (Terry and Wright 2005; Danton et al. 2003; Albery et al. 2000) New Zealand, (Hammond 2009) and Israel/US (LoParco et al. 2024). One US study found that the amount of alcohol consumed moderated relative attitudes (DACU was considered less dangerous than driving after five, but not fewer, drinks) (McCarthy et al. 2007). This sentiment appears to be common among samples of youth in Canada and the US, (McCarthy et al. 2007; Greene 2018; Grilly 1981; McKiernan and Fleming 2017; Colonna et al. 2021) as supported by three studies which reported differences by age (EKOS Research Associates Inc. 2017; Corporate Research Associates 2018; McDonald et al. 2021). Men and recent or frequent cannabis users were also more likely to hold this belief (EKOS Research Associates Inc. 2017; McDonald et al. 2021). Some researchers suggest that this difference may be partially explained by limited exposure to the dangers cannabis impaired driving (McKiernan and Fleming 2017) or lack of thought given to DACU (Danton et al. 2003). In contrast, messages about the dangers of alcohol-impaired driving are ubiquitous in these countries. Indeed, the need for information around how cannabis affects driving was expressed in four studies (Greene 2018; EKOS Research Associates Inc. 2017; Hammond 2009; Colonna et al. 2021). Due to these demographic differences, such messaging may be directed at youth and cannabis users.

DACU was considered to be as dangerous or more dangerous that driving after drinking alcohol in five studies on all (Malhotra et al. 2017; EKOS Research Associates Inc. 2017; McDonald et al. 2021; Woods-Fry et al. 2020) or some measures (Porath-Waller 2008). These studies were varied in regard to legal status of recreational or medical cannabis. Driving under the combined influence of cannabis and alcohol was universally thought to be riskier than DACU alone across studies from Canada, the US, and Australia (Greene 2018; Swift et al. 2010; Fischer et al. 2006; Lenné et al. 2001; Porath-Waller 2008; Aitken et al. 2000).

### The relationship between legal status of recreational cannabis and attitudes toward DACU is unclear

Fifteen studies from the US (*n* = 10, 2016–2024) and Canada (*n* = 5, 2017–2021) compared attitudes toward DACU in participants surveyed before versus after legalization of recreational cannabis (*n* = 4) or in participants residing in US states with versus without legalized recreational cannabis (*n* = 7), reported anticipated changes in DACU following legalization (*n* = 2), or discussed the impact of legalization on risk perception (*n* = 2). In the US, where recreational cannabis was legalized at the state level, drivers were more likely to view DACU as problematic after legalization (Eichelberger 2016). Residents in states with established recreational cannabis retail markets were more likely to believe that DACU is risky than residents in states without established retail markets (Lensch et al. 2020). Residents in states without legalized recreational cannabis thought DACU was less safe, (Brown et al. 2022) expressed less support for per-se laws for legal blood-THC concentration limits, (Benedetti et al. 2021) and were less likely to endorse anti-legalization arguments related to potential increases in MVCs relative to those in states without (McGinty et al. 2017). Still, some of this research found no difference in attitude by state legal status (Otto et al. 2016; Wadsworth and Hammond 2018; Benedetti et al. 2021; LoParco et al. 2024). Otto et al. (Otto et al. 2016) did not find any association between legal status and DACU intention, willingness, control beliefs, norms, and DACU behaviour. Pre-post legalization comparisons of US data suggest that there were no changes in perceived safety of DACU, (Cuttler et al. 2018) perceived risk of impairment due to DACU, or perceived likelihood of arrest after legalization (Eichelberger 2019).

Five studies were conducted in Canada where recreational cannabis was legalized in October 2018. Prior to legalization, most participants believed that DACU prevalence would rise after recreational cannabis use became legal, (EKOS Research Associates Inc. 2017; Colonna et al. 2021) although most did not intend to DACU themselves (Corporate Research Associates 2018). Concerningly, participants in a remedial program for impaired drivers understood legalization to mean that DACU is a low-risk activity (Wickens et al. 2019). The belief that cannabis is less impairing than alcohol increased from pre- to two years post legalization (Woods-Fry et al. 2020).

Additionally, another study compared safety attitudes in youth from Canada, England, and the US (Wadsworth and Hammond 2019). At the time of study (2017) recreational cannabis use was prohibited in England, all but four US states, and Canada (although Canada had announced plans for legalization in 2018). Youth in England were significantly more likely to endorse that DACU increases the risk of accident by ‘a lot’ than youth in Canada or the US, while US youth were significantly less likely that Canadian youth. Overall, the available evidence from studies conducted between 2016 and 2024 suggests that legalization does not prompt positive attitudes toward DACU.

### Perceived risk of apprehension for DACU is low to moderate

Thirty-one studies from the US (*n* = 11 1998–2021), US and Israel (*n* = 1, 2024), Canada (*n* = 9, 2013–2023), Australia (*n* = 7, 2001–2023), England (*n* = 2, 2003–2005), and Canada, the US, and England (*n* = 1, 2019) reported on perceived risk of apprehension for DACU. With the exception of one US study, (Ward et al. 2017) participants in the US, (Ward et al. 2018; Greene 2018; Allen et al. 2016; Wadsworth and Hammond 2018; Wadsworth and Hammond 2019; AAA Foundation for Traffic Safety 2018; Eichelberger 2016; Townsend et al. 1996; Eichelberger 2019) Canada, (Wadsworth and Hammond 2019; EKOS Research Associates Inc. 2017; Corporate Research Associates 2018; Goodman et al. 2020; Jonah 2013; McKiernan and Fleming 2017; Colonna et al. 2021; Huỳnh et al. 2022) Australia, (Jones et al. 2006; Mills et al. 2023; Lenné et al. 2001; Matthews et al. 2014) and England (Wadsworth and Hammond 2019; Danton et al. 2003) perceived low to moderate risk of apprehension and/or penalty for DACU. Perceptions of apprehension and/or penalty for driving after drinking alcohol were higher than for DACU (AAA Foundation for Traffic Safety 2018; Eichelberger 2016; Goodman et al. 2020; Jonah 2013; Lenné et al. 2001; McDonald et al. 2021; Terry and Wright 2005; Wickens et al. 2023; Matthews et al. 2014; LoParco et al. 2024). When asked about the likelihood of being stopped by police while DACU, one to two-thirds (38–69%) of participants in seven studies agreed that they would probably not be stopped (Ward et al. 2018; Allen et al. 2016; Wadsworth and Hammond 2018; Wadsworth and Hammond 2019; Corporate Research Associates 2018; AAA Foundation for Traffic Safety 2018; Goodman et al. 2020). Participants in four Canadian and US studies acknowledged that determining cannabis-related impairment would be difficult for law enforcement (EKOS Research Associates Inc. 2017; McKiernan and Fleming 2017; Colonna et al. 2021; LoParco et al. 2024). Perceived likelihood of being caught for DACU was low among samples of youth in five studies, (Greene 2018; Lenné et al. 2001; Colonna et al. 2021; Danton et al. 2003; Townsend et al. 1996) although two studies reported greater concerns among younger participants (EKOS Research Associates Inc. 2017) or no relationship between age and perceived likelihood of legal consequences (Jonah 2013).

Support for various preventive policy options, including per-se laws for legal blood-THC limits and stricter penalties for offences, was expressed in studies from Canada (EKOS Research Associates Inc. 2017; Jonah 2013; Colonna et al. 2021) and the US (Benedetti et al. 2021; AAA Foundation for Traffic Safety 2018). Participants in all countries agreed that increasing the likelihood of detection (e.g., through random testing) and punishment would have a deterrent effect on DACU, (Swift et al. 2010; Corporate Research Associates 2018; Terry and Wright 2005) although the majority in one Australian study disagreed because they thought they would ‘pass roadside performance tests anyway’ (Lenné et al. 2001). A small majority of Australian medical cannabis users (56–65%) indicated that random drug testing deters them from DACU (Arkell et al. 2020b; Arkell et al. 2023a). However, evidence in support of such strategies is mixed. In two studies from the US and Australia, cannabis users with a history of DACU reported less worry about being charged for DACU (OR = 1.17) (Berg et al. 2018) and more willingness to drive if there was no chance of detection and punishment (Jones et al. 2006). Another study found no association between perceived risk of arrest and DACU (Eichelberger 2019). Studies were heterogeneous in terms of legal status of recreational and medical cannabis at the time the research was done and legal status appears unrelated to support for preventive policies. However, legal status and drug-driving laws vary widely across US states, and no additional US studies contrasted perceived apprehension risk on this basis.

## Discussion

This review examined attitudes toward DACU based on results from 70 studies. In 35 studies, attitudes toward DACU were predominantly negative (e.g., DACU is dangerous, affects driving ability, and increases crash risk). However, positive attitudes were predominantly expressed in 20 studies. Surprisingly, subsets of participants in 16 studies reported that cannabis actually improves or positively affects driving ability. Youth, men, and frequent cannabis users tended to view DACU more favorably than older participants, women, and occasional or non-users. There appear to be gaps in beliefs regarding personal v.s., others’ attitudes toward DACU; participants believed that, relative to others, they held less favorable attitudes toward DACU, had less intention to DACU, and would be less impaired or likely to have an accident when DACU.

Despite a dearth of longitudinal studies linking current attitudes to future engagement in DACU, 26 studies in this review showed associations between attitudes and past DACU, intended DACU, or willingness to DACU. This evidence aligns with theoretical models that connect evaluations of behavior to actual behavioural engagement, including the theories of Planned Behaviour, Reasoned Action, (Fishbein and Ajzen 1975) and the Prototype Willingness Model (Gerrard et al. 2008). Broadly, these models assert that future behaviours are predicted by a system of cascading beliefs whereby attitudes shape intention and/or willingness to perform a behavior, which in turn predicts behavioural engagement. Dispelling misunderstandings about DACU safety may therefore decrease its incidence. Current evidence supports drug-driving interventions aimed at changing attitudes and improving knowledge about associated risks (Razaghizad et al. 2021).

DACU is generally viewed more favorably than driving after alcohol use and this perception may make DACU more likely. As such, it may be tempting to develop public safety campaigns against DACU that frame the two activities as equally dangerous. However, this may not be advisable as experimental and epidemiological evidence has consistently demonstrated that driving after alcohol use is more dangerous than DACU. In driver simulator studies, cannabis has been shown to affect driving performance to an extent similar to blood alcohol concentration of 0.04–0.06% (Simmons et al. 2022). The risk of MVC is higher among drinking drivers than in those who used cannabis (Sewell et al. 2009; Drummer et al. 2020; Brubacher et al. 2019) and alcohol-related collisions are more likely to result in serious injury than cannabis-related collisions (Brubacher et al. 2023). Preliminary evidence suggests that use of THC-containing cannabis has a negligible impact on driving performance when used for medical purposes (Arkell et al. 2023b; Arkell et al. 2021; Manning et al. 2024). Nonetheless, cannabis use does pose an MVC risk, and it is concerning that many people are unaware of this risk. Public education is needed to correct misconceptions about DACU safety, without emphasizing its risk relative to alcohol. Reassuringly, our results show that most people believe co-consumption of alcohol and cannabis is more detrimental to driving performance than consumption of either substance alone. This aligns with evidence from experimental driving studies (Simmons et al. 2022).

Legalization of recreational cannabis appeared to have an inconsistent effect on perceptions of DACU. One explanation is that legal changes influence cultural norms gradually, as most studies were conducted shortly before and after legalization. Further, differences in country and state-level dialogue on the risks of DACU around the time of legalization may have obfuscated the impact of legalization on attitudes.

Results of this analysis show that perceived risk of apprehension for DACU is low. In accordance with Deterrence Theory, enforcement agencies and policy makers seek to increase perception of certain, swift and severe punishment for illegal driving behavior (Bates et al. 2012; Davey and Freeman 2011; Hasan et al. 2022). Problematically, populations who tend to exhibit relatively positive attitudes toward DACU, including younger drivers and recent cannabis users are less likely to believe they will be caught by police. One reviewed study found that perceived likelihood of legal consequences is inversely associated with willingness to DACU among recent cannabis users (Jones et al. 2006). As such, policies that facilitate detection (e.g., roadside oral fluid testing) are encouraged, especially when directed at at-risk populations.

Currently, country-level impaired driving laws and detection approaches vary. Australia has a zero-tolerance policy, Canada has a zero-tolerance policy for young, novice, and commercial drivers but introduced per-se limits of THC in blood of ≥ 2 and 5ng/mL for other drivers in 2018, (Department of Justice Canada 2021) and the US has a mix of zero-tolerance, per-se, under the influence, and permissible inference laws (Conference and of State Legislatures.Marijuana-Impaired Driving 2023). Canada, Australia, and some US states permit oral fluid roadside testing, but uptake is variable. The state of Victoria has operated a high-visibility random oral fluid screening program since 2004, which has resulted in behavioural change, presumably because public awareness increased perceived likelihood of detection (Boorman and Owens 2009; Cameron et al. 2022). In contrast, in Canada and US states with authorization to collect oral fluid, police must have reasonable grounds to suspect DACU before they may demand a sample blood for testing. As THC blood levels drop rapidly after smoking cannabis, delays as police gather evidence may result in THC levels falling below per se limits before blood samples are obtained. Legal regimes that include visible enforcement along with well-publicized media campaigns can enhance deterrent effects by making drivers aware of policing efforts and legal penalties (Davey and Freeman 2011).

Educating drivers on the risks of DACU is strongly encouraged, but attitudinal change alone may not be sufficient to prevent this behavior. Even drivers who believe that cannabis impairs their ability to drive safely report driving within one hour of cannabis use (Cuttler et al. 2018). Behaviour is influenced by many factors including attitudes, norms, perceived behavioural control, and expectations. Five included studies found that DACU is predicted by perceived behavioural control (i.e., beliefs about ability to engage or not engage in DACU), descriptive norms (i.e., the extent to which DACU is believed to be common or normal), and injunctive norms (i.e., the extent to which others approve or disapprove of DACU) over and above attitudes (Earle et al. 2020; Ward et al. 2018; Otto et al. 2016; Scott et al. 2021). Consistent with these findings, several other studies report associations between perceived control, norms, and actual or intended DACU (McCarthy et al. 2007; Aston et al. 2016; Porath-Waller 2008; Aitken et al. 2000; Berg et al. 2018; Colonna et al. 2021; Huỳnh et al. 2022). Although not the focus of this review, these malleable social-cognitive antecedents of DACU behavior provide opportunities for intervention (Miller and Prentice 2016).

The studies included in this review largely focused on between-person differences in attitudes as predictors of DACU. However, individuals may decide to DACU on some occasions while deciding against it on others. Sometimes these decisions are made while under the influence of cannabis. Users who report feeling high are more likely to report that they can safely DACU than those who are not high (Allen et al. 2016). A similar pattern has been observed in regard to perceptions of danger and willingness to drive after drinking alcohol (Morris et al. 2014; Quinn and Fromme 2012). Given the importance of intoxication to one’s ability to make safer decisions, research on event-level characteristics of DACU decision-making is encouraged. Similar to the ‘arrive alive’ public service announcements for impaired driving campaigns, (CNW Group 2018) cannabis campaigns might encourage people to identify designated drivers and decide how they will be getting home after cannabis consumption before using.

### Strengths and limitations

This review has several strengths. To our knowledge, this is the first systematic review of attitudes toward DACU. It is comprised of 70 studies from seven countries that sampled various populations, including recreational and medical cannabis users, youth, and drivers with a history of DACU. This review also has some limitations. Interpretation of the present findings may be limited due to substantial heterogeneity between studies (e.g., variation in study design, number of participants, use of different questions). Attitudes were assessed in a variety of ways (e.g., dangerousness of DACU, effects of cannabis on crash risk or driving performance). The terminology used in survey studies may have implied varying degrees of impairment. For example, ‘driving under the influence of cannabis’ or ‘driving while impaired by cannabis’ may have biased respondents to endorse the dangerousness of DACU. The use of neutral language, such as ‘driving after using cannabis’ is encouraged, although a time interval may need to be specified and additional probes about ‘feeling the effects of cannabis’ might be useful. The questions posed usually referred to driving after using cannabis without specifying time elapsed from using cannabis to driving, dose, THC/CBD composition, or route of administration, leaving these parameters to participants’ interpretation. Consuming greater quantities of cannabis or cannabis with higher THC content may affect driving performance more dramatically, and the route of administration may affect the magnitude and duration of potential impairment (Burt et al. 2021). Further work could explore how attitudes toward DACU vary with dose, THC/CBD composition, route of administration, or time since use. Despite differences in methodology and outcomes measured, included studies converged on six broad themes that offer direction for intervention efforts.

## Conclusions

The current review revealed that the majority of participants in most studies consider DACU to be unsafe. That said, more education is needed as a minority of people do not perceive DACU as dangerous and individuals with a history of DACU tended to express willingness to DACU in the future. As legalization of recreational cannabis expands, concerns about what constitutes safer consumption or practices which mitigate harms to oneself and others will also likely increase. Because DACU has the potential to jeopardize the health and safety of not only the driver but also other road users, it will be crucial to consider attitudes toward DACU, how they vary across subpopulations, and how they can be changed to promote safer driving practices. This review has implications for campaigns to prevent DACU which include messaging to increase perception of risk and certainty of apprehension.

## Supplementary Information

Additional file 1. Search Strategy.

Additional file 2. Study Summary Table.

Additional file 3. Thematic synthesis codes.

## Data Availability

The datasets used and/or analyzed during the current study are available from the corresponding author on reasonable request.

## References

[CR1] AAA Foundation for Traffic Safety. 2018 Traffic Safety Culture Index. Washington (DC): AAA Foundation for Traffic Safety; 2019.

[CR2] Adams K, Smith L, Hind N. Drug driving among police detainees in Australia. Trends Issues Crime Crim Justice. 2008;357:1–6.

[CR3] Aitken C, Kerger M, Crofts N. Drivers who use illicit drugs: behaviour and perceived risks. Drugs: Educ Prev Policy. 2000;7(1):39–50.

[CR4] Ajzen I. The theory of planned behavior. Organ Behav Hum Decis Process. 1991;50(2):179–211.

[CR5] Albery IP, Strang J, Gossop M, Griffiths P. Illicit drugs and driving: prevalence, beliefs and accident involvement among a cohort of current out-of-treatment drug users. Drug Alcohol Depend. 2000;58(1–2):197–204. 10669072 10.1016/s0376-8716(99)00101-5

[CR6] Allen JA, Davis KC, Duke JC, Nonnemaker JM, Bradfield BR, Farrelly MC, et al. Association between self-reports of being high and perceptions about the safety of drugged and drunk driving. Health Educ Res. 2016;31(4):535–41. 27142851 10.1093/her/cyw023

[CR7] Arkell TR, Vinckenbosch F, Kevin RC, Theunissen EL, McGregor IS, Ramaekers JG. Effect of cannabidiol and Δ9-tetrahydrocannabinol on driving performance: a randomized clinical trial. JAMA. 2020a;324(21):2177–86. 33258890 10.1001/jama.2020.21218 PMC7709000

[CR8] Arkell TR, Lintzeris N, Mills L, Suraev A, Arnold JC, McGregor IS. Driving-related behaviours, attitudes and perceptions among Australian medical cannabis users: results from the CAMS 18–19 survey. Accid Anal Prev. 2020b;148: 105784. 33017729 10.1016/j.aap.2020.105784

[CR9] Arkell TR, McCartney D, McGregor IS. Medical cannabis and driving. Aust J Gen Pract. 2021;50(6):357–62. 34059836 10.31128/AJGP-02-21-5840

[CR10] Arkell TR, Abelev SV, Mills L, Suraev A, Arnold JC, Lintzeris N, McGregor IS. Driving-related behaviors, attitudes, and perceptions among Australian medical cannabis users: results from the CAMS 20 survey. J Cannabis Res. 2023a;5(1):35. 37674243 10.1186/s42238-023-00202-y PMC10481606

[CR11] Arkell TR, Manning B, Downey LA, Hayley AC. A Semi-Naturalistic, Open-Label Trial Examining the Effect of Prescribed Medical Cannabis on Neurocognitive Performance. CNS Drugs. 2023b;37(11):981–92. 37945917 10.1007/s40263-023-01046-z PMC10667416

[CR12] Arterberry BJ, Treloar HR, Smith AE, Martens MP, Pedersen SL, McArthy DM. Marijuana use, driving, and related cognitions. Psychol of Addict Behav. 2013;27(3):854–60. 10.1037/a0030877 PMC3980451 23276319

[CR13] Arterberry BJ, Treloar H, McCarthy DM. Empirical profiles of alcohol and marijuana use, drugged driving, and risk perceptions. J Stud Alcohol Drugs. 2017;78(6):889–98. 29087824 10.15288/jsad.2017.78.889 PMC5668995

[CR14] Asbridge M, Hayden JA, Cartwright JL. Acute cannabis consumption and motor vehicle collision risk: systematic review of observational studies and meta-analysis. Br Med J. 2012;344: e536. 22323502 10.1136/bmj.e536 PMC3277079

[CR15] Aston ER, Merrill JE, McCarthy DM, Metrik J. Risk factors for driving after and during marijuana use. J Stud Alcohol Drugs. 2016;77(2):309–16. 26997189 10.15288/jsad.2016.77.309 PMC4803663

[CR16] Auguste ME, Zambrano VC. Self-reported impacts of recreational and medicinal cannabis use on driving ability and amount of wait time before driving. Traffic Inj Prev. 2023;24(3):237–41. 36787207 10.1080/15389588.2023.2172679

[CR17] Barrie LR, Jones SC, Wiese E. “At least I’m not drink-driving”: Formative research for a social marketing campaign to reduce drug-driving among young drivers. Australas Mark J. 2011;19(1):71–5.

[CR18] Bates L, Soole D, Watson B. The effectiveness of traffic policing in reducing traffic crashes. Policing Secur Pract. London: Palgrave Macmillan UK; 2012. p. 90–109.

[CR19] Beasley EE, Beirness DJ. Alcohol and Drug Use Among Drivers Following the Introduction of Immediate Roadside Prohibitions in British Columbia: Findings from the 2012 Roadside Survey. Ottawa: Canadian Centre on Substance Abuse; 2012.

[CR20] Beirness D. Alcohol and drug use by drivers in British Columbia: findings from the 2018 Roadside Survey. Victoria, British Columbia: RoadSafetyBC; 2018.

[CR21] Beirness D. 2022 Roadside survey of alcohol and drug use by drivers in Ontario: Final report.: The Ontario Ministry of Transportation; 2023.

[CR22] Beirness D, Beasley E, McClafferty K. Alcohol and drug use among drivers in Ontario: Findings from the 2017 roadside survey. Toronto, ON: Ontario Ministry of Transportation; 2017.

[CR23] Benedetti MH, Li L, Neuroth LM, Humphries KD, Brooks-Russell A, Zhu M. Demographic and policy-based differences in behaviors and attitudes towards driving after marijuana use: an analysis of the 2013–2017 Traffic Safety Culture Index. BMC Res Notes. 2021;14(1):1–226. 34082823 10.1186/s13104-021-05643-3 PMC8176701

[CR24] Berg CJ, Daniel CN, Vu M, Li J, Martin K, Le L. Marijuana use and driving under the influence among young adults: a socioecological perspective on risk factors. Subst Use Misuse. 2018;53(3):370–80. 28777692 10.1080/10826084.2017.1327979 PMC6088242

[CR25] Boorman M, Owens K. The Victorian legislative framework for the random testing drivers at the roadside for the presence of illicit drugs: an evaluation of the characteristics of drivers detected from 2004 to 2006. Traffic Inj Prev. 2009;10(1):16–22. 19214873 10.1080/15389580802542365

[CR26] Borodovsky JT, Marsch LA, Scherer EA, Grucza RA, Hasin DS, Budney AJ. Perceived safety of cannabis intoxication predicts frequency of driving while intoxicated. Prev Med. 2020;131: 105956. 31863787 10.1016/j.ypmed.2019.105956 PMC6942456

[CR27] Bosker WM, Kuypers KPC, Theunissen EL, Surinx A, Blankespoor RJ, Skopp G, et al. Medicinal Δ9-tetrahydrocannabinol (dronabinol) impairs on-the-road driving performance of occasional and heavy cannabis users but is not detected in Standard Field Sobriety Tests. Addiction. 2012;107(10):1837–44. 22553980 10.1111/j.1360-0443.2012.03928.x

[CR28] Brands B, Mann RE, Wickens CM, Sproule B, Stoduto G, Sayer GS, et al. Acute and residual effects of smoked cannabis: impact on driving speed and lateral control, heart rate, and self-reported drug effects. Drug Alcohol Depend. 2019;205: 107641. 31678833 10.1016/j.drugalcdep.2019.107641

[CR29] Brown T, Banz B, Schmitt R, Gaffney G, Milavetz G, Camenga D, et al. A study of self-reported personal cannabis use and state legal status and associations with engagement in and perceptions of cannabis-impaired driving. Traffic Inj Prev. 2022;23(sup1):S183–6. 37014194 10.1080/15389588.2022.2124803 PMC10618935

[CR30] Brubacher JR, Chan H, Erdelyi S, Macdonald S, Asbridge M, Mann RE, et al. Cannabis use as a risk factor for causing motor vehicle crashes: a prospective study. Addiction. 2019;114(9):1616–26. 31106494 10.1111/add.14663 PMC6771478

[CR31] Brubacher JR, Chan H, Erdelyi S, Staples JA, Asbridge M, Mann RE. Cannabis legalization and detection of tetrahydrocannabinol in injured drivers. N Engl J Med. 2022;386(2):148–56. 35020985 10.1056/NEJMsa2109371

[CR32] Brubacher JR, Chan H, Erdelyi S, Yuan Y, Daoust R, Vaillancourt C, et al. High-‘n’-dry? A comparison of cannabis and alcohol use in drivers presenting to hospital after a vehicular collision. Addiction. 2023;118(8):1507–16. 36898848 10.1111/add.16186

[CR33] Burt TS, Brown TL, Milavetz G, McGehee DV. Mechanisms of cannabis impairment: implications for modeling driving performance. Forensic Sci Int. 2021;328: 110902. 34634690 10.1016/j.forsciint.2021.110902

[CR34] Cameron M, Newstead S, Clark B, Thompson L. Evaluation of an increase in roadside drug testing in Victoria based on models of the crash effects of random and targeted roadside tests. J Road Safety. 2022;33(2):17–32.

[CR35] Canada Go. Canadian cannabis survey 2018. 2018.

[CR36] Cavazos-Rehg PA, Krauss MJ, Sowles SJ, Zewdie K, Bierut L. Operating a motor vehicle after marijuana use: perspectives from people who use high-potency marijuana. Subst Abus. 2018;39(1):21–6. 28799883 10.1080/08897077.2017.1365802

[CR37] CNW Group. 30th 'arrive alive, drive sober' campaign launches ahead of cannabis legalization. Canadian NewsWire. 2018 May 8.

[CR38] Colonna R, Hand CL, Holmes JD, Alvarez L. Exploring youths’ beliefs towards cannabis and driving: a mixed method study. Transp Res Part F Traffic Psychol Behav. 2021;82:429–39.

[CR39] Corporate Research Associates. 2018 Edmonton and Area Traffic Safety Culture Survey. Edmonton (AB): City of Edmonton, Traffic Safety Section; 2019.

[CR40] Covidence systematic review software. 2023. Veritas Health Innovation, Melbourne, Australia. Available at www.covidence.org.

[CR41] Cuttler C, Sexton M, Mischley LK. Driving under the influence of cannabis: an examination of driving beliefs and practices of medical and recreational cannabis users across the United States. Cannabis. 2018;1(2):1–14.

[CR42] Danton K, Misselke L, Bacon R, Done J. Attitudes of young people toward driving after smoking cannabis or after drinking alcohol. Health Educ J. 2003;62(1):50–60.

[CR43] Davey JD, Freeman JE. Improving Road Safety through Deterrence-Based Initiatives: A review of research. Sultan Qaboos Univ Med J. 2011;11(1):29–37. PMC3074684 21509205

[CR44] Davis KC, Allen J, Duke J, Nonnemaker J, Bradfield B, Farrelly MC, et al. Correlates of marijuana drugged driving and openness to driving while high: evidence from Colorado and Washington. PLoS ONE. 2016;11(1): e0146853. 26800209 10.1371/journal.pone.0146853 PMC4723241

[CR45] Department of Justice Canada. Legislative background: reforms to the Transportation Provisions of the Criminal Code (Bill C-46) [Internet]: Department of Justice Canada; 2021 [cited 2023 Dec 1]. Available from: https://www.justice.gc.ca/eng/cj-jp/sidl-rlcfa/c46/p4.html#sec44.

[CR46] Downey LA, King R, Papafotiou K, Swann P, Ogden E, Boorman M, Stough C. The effects of cannabis and alcohol on simulated driving: influences of dose and experience. Accid Anal Prev. 2013;50:879–86. 22871272 10.1016/j.aap.2012.07.016

[CR47] Drummer OH, Gerostamoulos D, Di Rago M, Woodford NW, Morris C, Frederiksen T, et al. Odds of culpability associated with use of impairing drugs in injured drivers in Victoria. Australia Accid Anal Prev. 2020;135: 105389. 31812899 10.1016/j.aap.2019.105389

[CR48] Duckworth JC, Lee CM. Associations among simultaneous and co-occurring use of alcohol and marijuana, risky driving, and perceived risk. Addict Behav. 2019;96:39–42. 31030178 10.1016/j.addbeh.2019.03.019 PMC6579674

[CR49] Earle AM, Napper LE, LaBrie JW, Brooks-Russell A, Smith DJ, de Rutte J. Examining interactions within the theory of planned behavior in the prediction of intentions to engage in cannabis-related driving behaviors. J Am Coll Health. 2020;68(4):374–80. 30681931 10.1080/07448481.2018.1557197 PMC6658360

[CR50] Eichelberger AH. Survey of U.S. drivers about marijuana, alcohol, and driving. Arlington (VA): Insurance Institute for Highway Safety; 2016.

[CR51] Eichelberger AH. Marijuana use and driving in Washington State: risk perceptions and behaviors before and after implementation of retail sales. Traffic Inj Prev. 2019;20(1):23–9. 30822133 10.1080/15389588.2018.1530769

[CR52] Eichelberger A. Prevalence of alcohol, cannabis, and simultaneous use among drivers in six States. Transp Res Rec. 2023;2677(11):237–44.

[CR53] EKOS Research Associates Inc. Public opinion research on drug mpaired driving: baseline survey findings report. Ottawa (CA); 2017.

[CR54] Fink DS, Stohl M, Sarvet AL, Cerda M, Keyes KM, Hasin DS. Medical marijuana laws and driving under the influence of marijuana and alcohol. Addiction. 2020;115(10):1944–53. 32141142 10.1111/add.15031 PMC7483706

[CR55] Fischer B, Ivsins A, Rehm Jr, Webster CM, Rudzinski K, Rodopoulos J, Patra J. Factors associated with high-frequency cannabis use and driving among a multi-site sample of university students in Ontario. Can J Criminol Crim Justice. 2014;56(2):185–200.

[CR56] Fischer B, Rodopoulos J, Rehm J, Ivsins A. Toking and driving: characteristics of Canadian university students who drive after cannabis use-an exploratory pilot study. Drugs: Educ Prev Policy. 2006;13(2):179–87.

[CR57] Fishbein M, Ajzen I. Belief, attitude, intention, and behavior: an introduction to theory and research. Reading (MA): Addison-Wesley; 1975.

[CR58] Gerrard M, Gibbons FX, Houlihan AE, Stock ML, Pomery EA. A dual-process approach to health risk decision making: the prototype willingness model. Dev Rev. 2008;28(1):29–61.

[CR59] Ginsburg KR, Winston FK, Senserrick TM, Garcia-Espana F, Kinsman S, Quistberg DA, et al. National young-driver survey: teen perspective and experience with factors that affect driving safety. Pediatrics. 2008;121(5):e1391–403. 18450882 10.1542/peds.2007-2595

[CR60] Godin G, Kok G. The theory of planned behavior: a review of its applications to health-related behaviors. Am J Health Promot. 1996;11(2):87–98. 10163601 10.4278/0890-1171-11.2.87

[CR61] Goodman ES, Cesar L-T, David H. Risk perceptions of cannabis vs. alcohol-impaired driving among Canadian young people. Drugs: Educ Prev Policy. 2020;27(3):205–12.

[CR62] Greene KM. Perceptions of driving after marijuana use compared to alcohol use among rural American young adults. Drug Alcohol Rev. 2018;37(5):637–44. 29464852 10.1111/dar.12686 PMC6028284

[CR63] Grilly DM. People’s views on marijuana, other drugs & driving: an update. J Psychoact Drugs. 1981;13(4):377–9. 10.1080/02791072.1981.10471896 7338747

[CR64] Hackman CL, Knowlden AP. Theory of reasoned action and theory of planned behavior-based dietary interventions in adolescents and young adults: a systematic review. Adolesc Health Med Ther. 2014;5:101–14. 24966710 10.2147/AHMT.S56207 PMC4057331

[CR65] Hammond K. Drug driving in New Zealand: a survey of community attitudes, experience and understanding. NZ: New Zealand Drug Foundation; 2009.

[CR66] Hartman RL, Huestis MA. Cannabis effects on driving skills. Clin Chem. 2013;59(3):478–92. 23220273 10.1373/clinchem.2012.194381 PMC3836260

[CR67] Hartman RL, Brown TL, Milavetz G, Spurgin A, Pierce RS, Gorelick DA, et al. Cannabis effects on driving lateral control with and without alcohol. Drug Alcohol Depend. 2015;154:25–37. 26144593 10.1016/j.drugalcdep.2015.06.015 PMC4536116

[CR68] Hasan R, Watson B, Haworth N, Oviedo-Trespalacios O. A systematic review of factors associated with illegal drug driving. Accid Anal Prev. 2022;168: 106574. 35152044 10.1016/j.aap.2022.106574

[CR69] Hausenblas HA, Carron AV, Mack DE. Application of the theories of reasoned action and planned behavior to exercise behavior: a meta-analysis. J Sport Exerc Psychol. 1997;19(1):36–51.

[CR70] Hultgren BA, Guttmannova K, Cadigan JM, Kilmer JR, Delawalla MLM, Lee CM, Larimer ME. Injunctive norms and driving under the influence and riding with an impaired driver among young adults in Washington State. J Adolesc Health. 2023a;73(5):852–8. 37530684 10.1016/j.jadohealth.2023.06.010 PMC11837866

[CR71] Huỳnh C, Beaulieu-Thibodeau A, Fallu JS, Bergeron J, Jacques A, Brochu S. Typologies of Canadian young adults who drive after cannabis use: a two-step cluster analysis. Behav Sci Law. 2022;40(2):310–30. 35445426 10.1002/bsl.2575

[CR72] Johnson MB, Kelley-Baker T, Voas RB, Lacey JH. The prevalence of cannabis-involved driving in California. Drug Alcohol Depend. 2012;123(1–3):105–9. 22101027 10.1016/j.drugalcdep.2011.10.023 PMC3755617

[CR73] Jonah B. CCMTA public opinion survey of drugs and driving in Canada. Ottawa (ON): Canadian Council of Motor Transport Administrators; 2013.

[CR74] Jones C, Donnelly N, Swift W, Weatherburn D, Jones C, Donnelly N, et al. Preventing cannabis users from driving under the influence of cannabis. Accid Anal Prev. 2006;38(5):854–61. 16574046 10.1016/j.aap.2006.02.010

[CR75] Kmet L, Lee RC, Cook MS. Standard quality assessment criteria for evaluating primary research papers from a variety of fields. Edmonton: Alberta Heritage Foundation for Medical Research; 2004.

[CR76] Kohn C, Saleheen H, Borrup K, Rogers S, Lapidus G. Correlates of drug use and driving among undergraduate college students. Traffic Inj Prev. 2014;15(2):119–24. 24345012 10.1080/15389588.2013.803221

[CR77] Lenné MG, Fry CLM, Dietze P, Rumbold G. Attitudes and experiences of people who use cannabis and drive: implications for drugs and driving legislation in Victoria. Australia Drugs: Educ Prev Policy. 2001;8(4):307–13.

[CR78] Lensch T, Sloan K, Ausmus J, Pearson JL, Clements-Nolle K, Goodman S, Hammond D. Cannabis use and driving under the influence: behaviors and attitudes by state-level legal sale of recreational cannabis. Prev Med. 2020;141: 106320. 33161068 10.1016/j.ypmed.2020.106320 PMC8083159

[CR79] Li MC, Brady JE, DiMaggio CJ, Lusardi AR, Tzong KY, Li G. Marijuana use and motor vehicle crashes. Epidemiol Rev. 2012;34(1):65–72. 21976636 10.1093/epirev/mxr017 PMC3276316

[CR80] Li G, Chihuri S, Brady JE. Role of alcohol and marijuana use in the initiation of fatal two-vehicle crashes. Ann Epidemiol. 2017;27(5):342–7. 28595738 10.1016/j.annepidem.2017.05.003

[CR81] LoParco CR, Cui Y, Bar-Zeev Y, Levine H, Duan Z, Wang Y, et al. Driving under the influence of cannabis versus alcohol: a mixed-methods study examining perceptions and related risk behaviors among US and Israeli adults. Addict Behav. 2024;148: 107843. 37660497 10.1016/j.addbeh.2023.107843 PMC10591998

[CR82] MacDonald S, Mann R, Chipman M, Pakula B, Erickson P, Hathaway A, MacIntyre P. Driving behavior under the influence of cannabis or cocaine. Traffic Inj Prev. 2008;9(3):190–4. 18570139 10.1080/15389580802040295

[CR83] Malhotra N, Starkey NJ, Charlton SG. Driving under the influence of drugs: perceptions and attitudes of New Zealand drivers. Accid Anal Prev. 2017;106:44–52. 28554064 10.1016/j.aap.2017.05.011

[CR84] Manning B, Arkell TR, Hayley AC, Downey LA. A semi-naturalistic open-label study examining the effect of prescribed medical cannabis use on simulated driving performance. J Psychopharmacol. 2024;0(0):02698811241229524. 10.1177/02698811241229524 PMC10944578 38332655

[CR85] Matthews AJ, Bruno R, Dietze P, Butler K, Burns L. Driving under the influence among frequent ecstasy consumers in Australia: trends over time and the role of risk perceptions. Drug Alcohol Depend. 2014;144:218–24. 25282306 10.1016/j.drugalcdep.2014.09.015

[CR86] McCarthy DM, Lynch AM, Pederson SL. Driving after use of alcohol and marijuana in college students. Psychol Addict Behav. 2007;21(3):425–30. 17874895 10.1037/0893-164X.21.3.425

[CR87] McCartney D, Arkell TR, Irwin C, McGregor IS. Determining the magnitude and duration of acute Δ9-tetrahydrocannabinol (Δ9-THC)-induced driving and cognitive impairment: a systematic and meta-analytic review. Neurosci Biobehav Rev. 2021;126:175–93. 33497784 10.1016/j.neubiorev.2021.01.003

[CR88] McDonald AJ, Hamilton HA, Wickens CM, Watson TM, Elton-Marshall T, Wardell JD, et al. Driving under the influence of cannabis risk perceptions and behaviour: a population-based study in Ontario Canada. Prev Med. 2021;153: 106793. 34517043 10.1016/j.ypmed.2021.106793

[CR89] McGinty EE, Niederdeppe J, Heley K, Barry CL. Public perceptions of arguments supporting and opposing recreational marijuana legalization. Prev Med. 2017;99:80–6. 28189806 10.1016/j.ypmed.2017.01.024

[CR90] McHugh ML. Interrater reliability: the kappa statistic. Biochem Med. 2012;22(3):276–82. PMC3900052 23092060

[CR91] McKiernan A, Fleming K. Canadian youth perceptions on cannabis. Ottawa (ON): Canadian Centre on Substance Abuse; 2017.

[CR92] Micallef J, Dupouey J, Jouve E, Truillet R, Lacarelle B, Taillard J, et al. Cannabis smoking impairs driving performance on the simulator and real driving: a randomized, double-blind, placebo-controlled, crossover trial. Fundam Clin Pharmacol. 2018;32(5):558–70. 29752828 10.1111/fcp.12382

[CR93] Miller R, Brown T, Schmitt R, Gaffney G, Milavetz G. Predicting changes in driving performance in individuals who use cannabis following acute use based on self-reported readiness to drive. Accident analysis and prevention. 2024;195:107376-. 10.1016/j.aap.2023.107376 37984112

[CR94] Miller DT, Prentice DA. Changing norms to change behavior. Annu Rev Psychol. 2016;67(1):339–61. 26253542 10.1146/annurev-psych-010814-015013

[CR95] Mills L, Freeman J. Investigating predictors of driving immediately after consuming cannabis: a study of medical and recreational cannabis users in Australia. Transp Res f: Traffic Psychol Behav. 2023;96:213–21.

[CR96] Mills L, Freeman J, Rowland B. Australian daily cannabis users’ use of police avoidance strategies and compensatory behaviours to manage the risks of drug driving. Drug Alcohol Rev. 2023;42(6):1577–86. 37323052 10.1111/dar.13705

[CR97] Morris DH, Treloar HR, Niculete ME, McCarthy DM. Perceived danger while intoxicated uniquely contributes to driving after drinking. Alcohol Clin Exp Res. 2014;38(2):521–8. 24033630 10.1111/acer.12252 PMC3866225

[CR98] National Conference of State Legislatures. Marijuana-Impaired Driving. 2023.

[CR99] Ortiz-Peregrina S, Casares-López M, Ortiz C, Castro-Torres JJ, Martino F, Jiménez JR. Comparison of the effects of alcohol and cannabis on visual function and driving performance. Does the visual impairment affect driving? Drug Alcohol Depend. 2022;237:109538. 10.1016/j.drugalcdep.2022.109538 35717788

[CR100] Otto J, Finley K, Ward NJ. An assessment of traffic safety culture related to driving after cannabis use. Helena (MT): Center for Health and Safety Culture, Western Transportation Institute, Montana State University; 2016 2016 Dec.

[CR101] Page MJ, McKenzie JE, Bossuyt PM, Boutron I, Hoffmann TC, Mulrow CD, et al. The PRISMA 2020 statement: an updated guideline for reporting systematic reviews. J Clin Epidemiol. 2021;134:178–89. 33789819 10.1016/j.jclinepi.2021.03.001

[CR102] Pino MJ, Herruzo C, Raya A, Herruzo J. Legal and illegal substance consumption and traffic accident risk perception among Spanish young people. Soc Indic Res. 2016;129(2):835–45.

[CR103] Porath-Waller AJ. Driving high: a study of student driving behaviours and attitudes towards marijuana use and driving [thesis]: Ottawa (ON): Carelton University; 2008; 2008.

[CR104] Quinn PD, Fromme K. Event-level associations between objective and subjective alcohol intoxication and driving after drinking across the college years. Psychol Addict Behav. 2012;26(3):384–92. 21688876 10.1037/a0024275 PMC3260341

[CR105] Ramaekers JG, Robbe HW, O’Hanlon JF. Marijuana, alcohol and actual driving performance. Hum Psychopharmacol. 2000;15(7):551–8. 12404625 10.1002/1099-1077(200010)15:7<551::AID-HUP236>3.0.CO;2-P

[CR106] Razaghizad A, Windle SB, Gore G, Benedetti A, Ells C, Grad R, et al. Interventions to prevent drugged driving: a systematic review. Am J Prev Med. 2021;61(2):267–80. 34099354 10.1016/j.amepre.2021.03.012

[CR107] Rogeberg O, Elvik R. The effects of cannabis intoxication on motor vehicle collision revisited and revised. Addiction. 2016;111(8):1348–59. 26878835 10.1111/add.13347

[CR108] Ronen A, Gershon P, Drobiner H, Rabinovich A, Bar-Hamburger R, Mechoulam R, et al. Effects of THC on driving performance, physiological state and subjective feelings relative to alcohol. Accid Anal Prev. 2008;40(3):926–34. 18460360 10.1016/j.aap.2007.10.011

[CR109] Ryan C, Hesselgreaves H, Wu O, Paul J, Dixon-Hughes J, Moss JG. Protocol for a systematic review and thematic synthesis of patient experiences of central venous access devices in anti-cancer treatment. Syst Rev. 2018;7(1):61. 29669583 10.1186/s13643-018-0721-x PMC5907379

[CR110] Scott B, Ward N, Otto J, Finley K. Modeling the system of beliefs that influence driving under the influence of cannabis (DUIC) in Washington State. Accid Anal Prev. 2021;151: 105988. 33484972 10.1016/j.aap.2021.105988

[CR111] Sewell RA, Poling J, Sofuoglu M. The effect of cannabis compared with alcohol on driving. Am J Addict. 2009;18(3):185–93. 19340636 10.1080/10550490902786934 PMC2722956

[CR112] Shults RA, Elder RW, Sleet DA, Nichols JL, Alao MO, Carande-Kulis VG, et al. Reviews of evidence regarding interventions to reduce alcohol-impaired driving. Am J Prev Med. 2001;21(4S):66–88. 11691562 10.1016/s0749-3797(01)00381-6

[CR113] Simmons SM, Caird JK, Sterzer F, Asbridge M. The effects of cannabis and alcohol on driving performance and driver behaviour: a systematic review and meta-analysis. Addiction. 2022;117(7):1843–56. 35083810 10.1111/add.15770

[CR114] Swift W, Jones C, Donnelly N. Cannabis use while driving: a descriptive study of Australian cannabis users. Drugs Educ Prev Policy. 2010;17(5):573–86.

[CR115] Teeters JB, King SA, Hubbard SM. A mobile phone-based brief intervention with personalized feedback and interactive text messaging is associated with changes in driving after cannabis use cognitions in a proof-of-concept pilot trial. Exp Clin Psychopharmacol. 2021;29(2):203–9. 34043401 10.1037/pha0000442 PMC8376090

[CR116] Terry P, Wright KA. Self-reported driving behaviour and attitudes towards driving under the influence of cannabis among three different user groups in England. Addict Behav. 2005;30(3):619–26. 15718082 10.1016/j.addbeh.2004.08.007

[CR117] Townsend TN, Lane J, Dewa CS, Brittingham AM. Driving after drug or alcohol use: findings from the 1996 National Household Survey on Drug Abuse. Washington (DC): Dept. of Health and Human Services, Substance Abuse and Mental Health Services Administration; 1998.

[CR118] Wadsworth E, Hammond D. Differences in patterns of cannabis use among youth: prevalence, perceptions of harm and driving under the influence in the USA where non-medical cannabis markets have been established, proposed and prohibited. Drug Alcohol Rev. 2018;37(7):903–11. 29992695 10.1111/dar.12842 PMC6215732

[CR119] Wadsworth E, Hammond D. International differences in patterns of cannabis use among youth: prevalence, perceptions of harm, and driving under the influence in Canada. Engl U S Addict Behav. 2019;90:171–5. 10.1016/j.addbeh.2018.10.050 PMC6324962 30412908

[CR120] Ward NJ, Otto J, Schell W, Finley K, Kelley-Baker T, Lacey JH. Cultural predictors of future intention to drive under the influence of cannabis (DUIC). Transp Res Part F Traffic Psychol Behav. 2017;49:215–25.

[CR121] Ward NJ, Schell W, Kelley-Baker T, Otto J, Finley K. Developing a theoretical foundation to change road user behavior and improve traffic safety: driving under the influence of cannabis (DUIC). Traffic Inj Prev. 2018;19(4):358–63. 29337600 10.1080/15389588.2018.1425548

[CR122] Wechsler H, Rohman M, Kotch JB, Idelson RK. Alcohol and other drug use and automobile safety: a survey of Boston-area teen-agers. J Sch Health. 1984;54(5):201–3. 6564306 10.1111/j.1746-1561.1984.tb08817.x

[CR123] Wickens CM, Watson TM, Mann RE, Brands B. Exploring perceptions among people who drive after cannabis use: collision risk, comparative optimism and normative influence. Drug Alcohol Rev. 2019;38(4):443–51. 30896069 10.1111/dar.12923

[CR124] Wickens CM, McDonald AJ, Stoduto G, Di Ciano P, Hamilton HA, Elton-Marshall T, et al. Risk perceptions of driving under the influence of cannabis: comparing medical and non-medical cannabis users. Transp Res f: Traffic Psychol Behav. 2023;95:36–45.

[CR125] Woods-Fry H, Robertson RD, Vanlaar WGM. Road Safety Monitor 2020: trends in marijuana use among Canadian drivers. Ottawa (ON): Traffic Injury Research Foundation; 2020.

