# Supplementary material for: Attitudes toward driving after cannabis use: a systematic review
Source: J Cannabis Res. 2024 Sep 28;6:37. doi: 10.1186/s42238-024-00240-0 (PMC11439277; doi:10.1186/s42238-024-00240-0)
Supplement: Supplementary file 1 — Additional file 1. Search Strategy. [file 42238_2024_240_MOESM1_ESM.docx]

**Supplementary File 1 - Search Strategy and Results**

Search date: May 31, 2022

**EMBASE (Ovid)**

| **Concept** | **Search term(s)** |
| --- | --- |
| Concept 1: cannabis use | 1. exp “cannabis use”/ |
|  | 2 . exp cannabinoid/ |
|  | 3. exp cannabis/ |
|  | 4. exp cannabis smoking/ |
|  | 5 . exp medical cannabis/ |
|  | 6 . exp tetrahydrocannabinol/ |
|  | 7. (cannabi* or hashish or mari#uana or THC or tetrahydrocannabinol or drug*).kw. |
|  | *1 or 2 or 3 or 4 or 5 or 6 or 7* |
| Concept 2: attitude | 1. exp attitude/ |
|  | 2. *perception/ |
|  | 3. exp risk perception/ |
|  | 4. exp social attitude/ |
|  | 5. exp risk attitude/ |
|  | 6. exp health belief/ |
|  | 7. (attitude* or percept* or belie* or risk or intention* or motiv* or expect* or willing* or opinion* or determin* or factor* or characteristic* or correlat*).kw. |
|  | *1 or 2 or 3 or 4 or 5 or 6 or 7* |
| Concept 3: driving ability | 1. exp motor vehicle/ |
|  | 2. driving ability/ |
|  | 3. car driving/ |
|  | 4. traffic accident/ |
|  | 5. exp road safety/ |
|  | 6. exp traffic safety/ |
|  | 7. exp vehicle safety/ |
|  | 8. exp passenger safety/ |
|  | 9. exp pedestrian safety/ |
|  | 10. (driv* or driving under the influence or DUIC or traffic or road).kw. |
|  | *1 or 2 or 3 or 4 or 5 or 6 or 7 or 8 or 9 or 10* |
| Concept 1 and concept 2 and concept 3: 106 | |

**PsycInfo (EBSCOhost)**

| **Concept** | **Search term(s)** |
| --- | --- |
| Concept 1: cannabis use | 1. SU cannabis |
|  | 2. SU cannabinoid* |
|  | 3. SU marijuana |
|  | 4. SU tetrahydrocannabinol |
|  | 5. SU hashish |
|  | *1 or 2 or 3 or 4 or 5* |
| Concept 2: attitude | 1. SU attitude* |
|  | 2. SU health attitude* |
|  | 3. SU “explicit attitude*” |
|  | 4. SU “implicit attitude*” |
|  | 5. SU “community attitude*” |
|  | 6. SU “public health attitude*” |
|  | 7. SU “drug usage attitude*” |
|  | 8. SU perception* OR SU perceive* |
|  | 9. SU “social perception*” |
|  | 10. SU “risk perception*” |
|  | 11. SU “risk taking” |
|  | 12. SU “risk assessment” |
|  | 13. SU intention |
|  | 14. SU “planned behavior” |
|  | 15. SU opinion |
|  | 16. belie* |
|  | 1. characteristic* |
|  | 1. correlat* |
|  | 1. factor* |
|  | *1 or 2 or 3 or 4 or 5 or 6 or 7 or 8 or 9 or 10 or 11 or 12 or 13 or 14 or 15 or 16* |
| Concept 3: driving ability | 1. driv* |
|  | 2. SU “driving behavior” |
|  | 3. SU “motor vehicle*” |
|  | 4. SU automobiles |
|  | 5. SU “driving under the influence” |
|  | 6. SU “transportation safety” |
|  | 7. SU transportation |
|  | 8. SU “motor traffic accidents” |
|  | 9. SU “accident proneness” |
|  | *1 or 2 or 3 or 4 or 5 or 6 or 7 or 8 or 9* |
| Concept 1 and concept 2 and concept 3: 216 | |

**Medline (Ovid)**

| **Concept** | **Search term(s)** |
| --- | --- |
| Concept 1: cannabis use | 1. exp "Marijuana Use"/ |
|  | 2. exp Cannabinoids/ |
|  | 3. Cannabis/ |
|  | 4. (cannabi* or hashish or mari#uana or THC or tetrahydrocannabinol).kw |
|  | *1 or 2 or 3 or 4* |
| Concept 2: attitude | 1. *Attitude/ |
|  | 2. *Perception/ |
|  | 3. *risk-taking/ |
|  | 4. intention/ |
|  | 5. *Motivation/ |
|  | 6. *Social Norms/ |
|  | 7. Social Perception/ |
|  | 1. Exp Peer Group/ |
|  | 9. *Health Knowledge, Attitudes, Practice/ |
|  | 10. (attitude* or percept* or belie* or expect* or understand* or willing* or opinion* or risk or determin* or correlat* or factor* or characteristic*).kw |
|  | *1 or 2 or 3 or 4 or 5 or 6 or 7 or 8 or 9 or 10* |
| Concept 3: driving ability | 1. Driving Under the Influence/ |
|  | 2. Automobile Driving/ |
|  | 3. exp Motor Vehicles/ |
|  | 4. Accidents, Traffic/ |
|  | 6. exp Dangerous Behavior/ |
|  | 5. (driv* or DUIC or traffic or road).kw |
|  | *1 or 2 or 3 or 4 or 5* |
| Concept 1 and concept 2 and concept 3: 40 | |

**TRID**

| **Concept** | **Search term(s)** |
| --- | --- |
| Concept 1: cannabis use | 1. cannabis OR cannabinoid* OR hashish OR marijuana OR marihuana OR THC OR tetrahydrocannabinol |
| Concept 2: attitude | 2. perceived OR perception* OR belie* OR attitude* OR opinion* OR motiv* OR intent* or expect* or understand* or willing* or determinant* or correlat* or characteristic* |
| Concept 3: driving | 3. driv* OR vehicle* OR car OR automobile* OR traffic OR road* or "driving under the influence" |
| Concept 1 and concept 2 and concept 3: 555 | |

Search date: Feb 26, 2024

**EMBASE (Ovid)**

| **Concept** | **Search term(s)** |
| --- | --- |
| Concept 1: cannabis use | 1.  exp “cannabis use”/ |
|  | 2 . exp cannabinoid/ |
|  | 3.  exp cannabis/ |
|  | 4.  exp cannabis smoking/ |
|  | 5 . exp medical cannabis/ |
|  | 6 . exp tetrahydrocannabinol/ |
|  | 7.  (cannabi* or hashish or mari#uana or THC or tetrahydrocannabinol or drug*).kw. |
|  | *1 or 2 or 3 or 4 or 5 or 6 or 7* |
| Concept 2: attitude | 1.  exp attitude/ |
|  | 2.  *perception/ |
|  | 3.  exp risk perception/ |
|  | 4.  exp social attitude/ |
|  | 5.  exp risk attitude/ |
|  | 6.  exp health belief/ |
|  | 7.  (attitude* or percept* or belie* or risk or intention* or motiv* or expect* or willing* or opinion* or determin* or factor* or characteristic* or correlat*).kw. |
|  | *1 or 2 or 3 or 4 or 5 or 6 or 7* |
| Concept 3: driving ability | 1. exp motor vehicle/ |
|  | 2. driving ability/ |
|  | 3. car driving/ |
|  | 4. traffic accident/ |
|  | 5. exp road safety/ |
|  | 6. exp traffic safety/ |
|  | 7. exp vehicle safety/ |
|  | 8. exp passenger safety/ |
|  | 9. exp pedestrian safety/ |
|  | 10. (driv* or driving under the influence or DUIC or traffic or road).kw. |
|  | *1 or 2 or 3 or 4 or 5 or 6 or 7 or 8 or 9 or 10* |
| Concept 1 and concept 2 and concept 3: 125 | |

**PsycInfo (EBSCOhost)**

| **Concept** | **Search term(s)** |
| --- | --- |
| Concept 1: cannabis use | 1. SU cannabis |
|  | 2. SU cannabinoid* |
|  | 3. SU marijuana |
|  | 4. SU tetrahydrocannabinol |
|  | 5. SU hashish |
|  | *1 or 2 or 3 or 4 or 5* |
| Concept 2: attitude | 1. SU attitude* |
|  | 2. SU health attitude* |
|  | 3. SU “explicit attitude*” |
|  | 4. SU “implicit attitude*” |
|  | 5. SU “community attitude*” |
|  | 6. SU “public health attitude*” |
|  | 7. SU “drug usage attitude*” |
|  | 8. SU perception* OR SU perceive* |
|  | 9. SU “social perception*” |
|  | 10. SU “risk perception*” |
|  | 11. SU “risk taking” |
|  | 12. SU “risk assessment” |
|  | 13. SU intention |
|  | 14. SU “planned behavior” |
|  | 15. SU opinion |
|  | 16. belie* |
|  | 1. characteristic* |
|  | 1. correlat* |
|  | 1. factor* |
|  | *1 or 2 or 3 or 4 or 5 or 6 or 7 or 8 or 9 or 10 or 11 or 12 or 13 or 14 or 15 or 16* |
| Concept 3: driving ability | 1. driv* |
|  | 2. SU “driving behavior” |
|  | 3. SU “motor vehicle*” |
|  | 4. SU automobiles |
|  | 5. SU “driving under the influence” |
|  | 6. SU “transportation safety” |
|  | 7. SU transportation |
|  | 8. SU “motor traffic accidents” |
|  | 9. SU “accident proneness” |
|  | *1 or 2 or 3 or 4 or 5 or 6 or 7 or 8 or 9* |
| Concept 1 and concept 2 and concept 3: 298 | |

**Medline (Ovid)**

| **Concept** | **Search term(s)** |
| --- | --- |
| Concept 1: cannabis use | 1. exp "Marijuana Use"/ |
|  | 2. exp Cannabinoids/ |
|  | 3. Cannabis/ |
|  | 4. (cannabi* or hashish or mari#uana or THC or tetrahydrocannabinol).kw |
|  | *1 or 2 or 3 or 4* |
| Concept 2: attitude | 1. *Attitude/ |
|  | 2. *Perception/ |
|  | 3. *risk-taking/ |
|  | 4. intention/ |
|  | 5. *Motivation/ |
|  | 6. *Social Norms/ |
|  | 7. Social Perception/ |
|  | 1. Exp Peer Group/ |
|  | 9. *Health Knowledge, Attitudes, Practice/ |
|  | 10. (attitude* or percept* or belie* or expect* or understand* or willing* or opinion* or risk or determin* or correlat* or factor* or characteristic*).kw |
|  | *1 or 2 or 3 or 4 or 5 or 6 or 7 or 8 or 9 or 10* |
| Concept 3: driving ability | 1. Driving Under the Influence/ |
|  | 2. Automobile Driving/ |
|  | 3. exp Motor Vehicles/ |
|  | 4. Accidents, Traffic/ |
|  | 6. exp Dangerous Behavior/ |
|  | 5. (driv* or DUIC or traffic or road).kw |
|  | *1 or 2 or 3 or 4 or 5* |
| Concept 1 and concept 2 and concept 3: 42 | |

**TRID**

| **Concept** | **Search term(s)** |
| --- | --- |
| Concept 1: cannabis use | 1. cannabis OR cannabinoid* OR hashish OR marijuana OR marihuana OR THC OR tetrahydrocannabinol |
| Concept 2: attitude | 2. perceived OR perception* OR belie* OR attitude* OR opinion* OR motiv* OR intent* or  expect* or understand* or willing* or determinant* or correlat* or characteristic* |
| Concept 3: driving | 3. driv* OR vehicle* OR car OR automobile* OR traffic OR road* or "driving under the influence" |
| Concept 1 and concept 2 and concept 3: 626 | |
