# Supplementary material for: Attitudes toward driving after cannabis use: a systematic review
Source: J Cannabis Res. 2024 Sep 28;6:37. doi: 10.1186/s42238-024-00240-0 (PMC11439277; doi:10.1186/s42238-024-00240-0)
Supplement: Supplementary file 2 — Additional file 2. Study Summary Table. [file 42238_2024_240_MOESM2_ESM.docx]

| **Author (year)** | **Country** | **N** | **Sample characteristics** | **Age M ± SD [range]** | **Sex (% male:% female)** | **Study design** | **Key findings** | **SQAC score** |  |
| --- | --- | --- | --- | --- | --- | --- | --- | --- | --- |
| Arkell et al (2023)[1] | Australia | 1,063 | General population; used cannabis for therapeutic reasons within the past 12 months and drove a motor vehicle in the past month. | 46.3 ± 13.5 [18+] | 56:43 | Online survey. | Non-medical cannabis was perceived to be more impairing for driving than medical cannabis; half (51%) thought that non-medical cannabis impairs driving ability and a minority (22.1%) thought that medical cannabis impairs driving ability. The majority (56%) indicated that roadside drug testing deters them from driving after using medical cannabis. Those who DACU were more likely to believe that recreational cannabis does not impair driving ability (OR=3.53) and were less deterred by roadside drug testing (OR=1.97). | 0.93 |  |
| Mills & Freeman (2023)[2] | Australia | 839 | General population; drivers who used cannabis within the past month. | 43.3 ± 13.12 [18-75] | 61:38 | Online survey. | Participants were asked to indicate the extent to which they 1) agreed that they ‘would be more likely to cause a crash’ if driving within 4 h of using cannabis (M=1.46 SD=0.75; 1 - strongly disagree to 4 - strongly agree); 2) ‘think driving soon after consuming cannabis should be considered an offence’ (M=2.5, SD=1.49; 1 - strongly disagree to 6 - strongly agree); and 3) ‘could drive safely soon after consuming cannabis’ (M=4.79, SD=1.34). Less than half (42%) said they would drive immediately after consuming cannabis (17% would wait 1 h, 8% 2 h, rest would wait between 3 and 12+ h).  *Sample may partially overlap with Mills, Freeman, & Rowland 2023 | 1 |  |
| Mills, Freeman & Rowland (2023)[3] | Australia | 487 | General population; drivers who used cannabis daily within the past month. | 43.79 ± 12.45 [18-75] | 58:61 | Online survey. | Most participants disagreed that cannabis reduces driving ability or MVC risk (M=1.31, SD=0.57; 1 – not at all to 4 – very much) and disagreed that they would be more likely to cause a crash when DACU (M=1.36, SD=0.66; 1 – strongly disagree to 4 – strongly agree). Participants considered being drug tested by police if they were to DACU (within 4 h of consumption) somewhat likely (M=3.35, SD=1.55; 1 – extremely unlikely to 6 – extremely likely). Frequency of weekly DACU was moderately associated with disagreeing that cannabis reduces driving ability (r=-0.219; p<.05).  *Sample may partially overlap with Mills & Freeman 2023 | 1 |  |
| Arkell et al (2020)[4] | Australia | 806 | General population; used cannabis for therapeutic reasons within the past 12 months and drove a motor vehicle within the past month. | 45 ± 13  [18+] | 58:42 | Online survey. | Most respondents (72%) agreed or strongly agreed that their medical cannabis use does not impair their driving. Respondents were 3% more likely to think that their DACU does not impair their driving for each additional day of cannabis use (p<.01). | 0.92 |  |
| Matthews et al (2014)[5] | Australia | 1,002 | Ecstasy users (at least monthly) who drove a car within the past 6 months. | [17-54] | 63:37 | Face-to-face interviews (Ecstasy and Related Drug Reporting System). | Low DACU-related crash risk perceptions were associated with DACU in the past 6 months (R²=.276, p<.001). | 1 |  |
| Barrie, Jones & Wiese (2011)[6] | Australia | NR; 6 focus groups with 6 - 8 people per group. | Young adults. | [18–25] | NR; 3 male-only and 3 female-only focus groups. | Focus groups. | Cannabis was thought to be safer than other illicit drugs or alcohol in the context of driving. Some believed that cannabis could improve driving performance by making the driver slow down. | 0.75 |  |
| Swift et al (2010)[7] | Australia | 320 | General population; licensed drivers who used cannabis within the past year. | 29  [18-73] Median = 26 | 68:32 | Face-to-face interviews. | Half (53%) believed DACU alone increases personal accident risk. Among cannabis users who DACU in the past year, a third (29%) felt their driving ability was impaired. | 0.95 |  |
| Adams et al (2008)[8] | Australia | 1,215 | Police detainees who participated in the Drug Use Monitoring in Australia program. | Past-year drug drivers = 29; non-drug drivers = 32 [18+] | 88:12 | Face-to-face interviews with police detainees in a police station or watch-house. | Most (68%) detainees believed cannabis never had an effect on their driving. “Cannabis was the most commonly reported drug that detainees perceived to have a positive effect (15%) or no effect (36%) on driving.” | 0.79 |  |
| Jones et al (2006)[9] | Australia | 320 | General population; used cannabis and drove within the past year. | 29  [18-73] Median = 26 | 67:33 | Face-to-face interviews. | Recent cannabis users indicated less willingness to drive in hypothetical scenarios with high v.s. low certainty of legal apprehension for DACU with random roadside drug tests (OR=.2, p<.001), but not high v.s. low punishment severity (OR=0.9, p=0.68). | 0.95 |  |
| Lenné et al (2001)[10] | Australia | 67 | Young cannabis users (used cannabis within the past month) who held a valid driver's license. | 23.6 ± 3.4  [18-25] | 45:55 | Interviews. | Over half (57%) indicated that DACU did not increase their accident risk. Nearly half (46%) believed that cannabis alone adversely affected their driving ability and a minority (12%) reported that cannabis improved their driving ability. | 0.83 |  |
| Aitken et al (2000)[11] | Australia | 8 | General population; drove (at least twice per week) who used cannabis for at least 3 years and used cannabis and alcohol concurrently at least twice per week. | 29.3  [19-48] | 25:75 | Focus groups. | Participants “unanimously perceived cannabis to be a very safe drug for driving”. | 0.89 (quantitative); 0.95 (qualitative) |  |
| Wickens et al (2023)[12] | Canada (Ontario) | 259 | General population; used cannabis within the past year. | [18+] | 62:38 | Telephone survey (Centre for Addiction and Mental Health Monitor). | Most respondents strongly agreed (52%) or somewhat agreed (25%) that DACU increases MVC risk. Most strongly agreed (37%) or somewhat agreed (18%) that DACU is safer than driving after drinking alcohol. Most strongly agreed (45%) or somewhat agreed (30%) that chances of getting caught by police are higher for driving after drinking alcohol than DACU. Among these, non-medical users were more likely to disagree that DACU increases MVC risk relative to medical/dual-purpose users (PR=1.61, p<.05). Medical/dual-purpose users were more likely than non-medical only users to agree that DACU is safer than driving after drinking alcohol (aPR=1.45, P<.05).  *Weighted sample (selection probabilities, regional representation, age and sex distribution based on ‘the most recently available Census’)  *Sample may partially overlap with McDonald et al 2021 | 1 |  |
| Huỳnh et al (2022)[13] | Canada | 910 | General population; licensed drivers who used cannabis within the past year. | 25.5 ± 5.4  [17-35] | 54:40 | Online survey. | Young adults who DACU did not share the same characteristics; 4 typologies were identified. "High frequent cannabis users who regularly DACU" were the least likely to perceive DACU as a risky behavior (compared with "well-adjusted youths with mild depressive-anxious symptoms", “high frequent cannabis users”, and “individuals with generalized deviance”. | 1 |  |
| Colonna et al (2021)[14] | Canada (Ontario) | 426 | Young drivers. | [18-24] | 47:53 | Online survey and focus group. | Participant beliefs regarding the dangers of DACU and risk of MVC were lower among drivers with a history of DACU v.s., those without and non-users. Qualitative themes included: “being high changes you; it is hard to tell if a driver is high; DACU is convenient, socially acceptable, and safe; legalization makes cannabis more attractive to use; and there is a need for testing, education, and prevention efforts that are relevant to youth”. Almost half (42%) of the sample expressed that “there is a chance they will engage in DACU in the future” (compared with 35% who reported having DACU). | 0.95 |  |
| McDonald et al (2021)[15] | Canada (Ontario) | 1,813 | General population. | [18+] | 46:54 | Telephone survey (Centre for Addiction and Mental Health Monitor). | Almost all (90%) of respondents agreed that DACU increases MVC risk. Most (82%) cannabis-using drivers that were not involved in DACU agreed that DACU increases MVC risk; a significantly smaller proportion (55%) of those who had DACU agreed. Disagreeing that DACU increases MVC risk was associated with past-year DACU (aOR=6.46, p<.001).  *Weighted sample (selection probabilities, regional representation, age and sex distribution based on ‘the most recently available Census’)  *Sample may partially overlap with Wickens et al 2023 | 1 |  |
| Goodman et al (2020)[16] | Canada | 870 | General population. | [16-30] | 48:52 | Online survey. | Almost one third (28%) of respondents believed that DACU “does not increase the risk of getting into an accident ‘at all’ or that it does so only ‘a little’”. Perceived risk of getting into an accident was negatively correlated with cannabis use status (ρ=.41, p<.001) and history of DACU (ρ= 0.27, p<.001). *Weighted sample (age, sex, region, population based on Statistics Canada Census 2017) | 1 |  |
| Woods-Fry et al (2020)[17] | Canada | 1,500 | General population; licensed drivers. | NR | NR | Online survey (Canadian Road Safety Monitor). | “The percentage of drivers who agreed that cannabis does not affect one’s driving as much as alcohol increased from 10.3% in 2018 to 13.4% in 2020 (z=2.46, p=.01), representing a 30% increase from pre- to 2-years-post legalization.” | 0.82 |  |
| Wadsworth & Hammond (2019)[18] | Canada, England & United States | 12,064 | Youth. | [16-19] | CA: 35:65 EN: 43:57 US: 40:60 | Online survey (International Tobacco Control Policy Evaluation Project Tobacco and Youth E-cigarette Survey). | “Approximately half of Canadian and English youth reported that driving within 2 h of using cannabis increased the risk of an accident by ‘a lot’” (52% and 57%, respectively), compared to less than half of United States youth (44%).  *Weighted sample (country, age, sex, ethnicity, and number of computers in home)  *Sample may partially overlap with Wadsworth & Hammond 2018 | 0.91 |  |
| Wickens et al (2019)[19] | Canada (Ontario) | 20 | Participants in a remedial program for impaired drivers who drove within the past year. | 32  [21-53] | 90:10 | Face-to-face interviews. | “Mixed perceptions regarding the dangerousness of DACU were expressed, with some participants denying increased collision risk except among novice cannabis users.” Participants demonstrated comparative optimism bias, expressed as lower estimates of personal MVC risk compared to others’ risk. “Many participants identified DACU as less risky than driving under the influence of alcohol or other drugs.” | 0.95 |  |
| Corporate Research Associates (2019)[20] | Canada (Alberta) | 2,006 | General population. | [16+] | 50:50 | Telephone survey. | Most (72%) participants perceived driving within 2 h of consuming cannabis as unacceptable. A majority (62%) agreed that a person who drives within 2 h of consuming cannabis would be impaired. Almost no drivers (3%) reported high likelihood of driving within 2 h after consuming cannabis once it becomes legal; most (80%) reported that they definitely will not. *Weighted sample (age, gender, and region based on Statistics Canada 2016 data) | 1 |  |
| EKOS Research Associates Inc. (2017)[21] | Canada | 2,132 | General population. | [16+] | 49:50 | Telephone and online survey. | Most respondents (81%) agreed that using cannabis impairs one's ability to drive or operate a vehicle and few (17%) stated that DACU poses no real risk.  *Weighted sample (age, gender and region based on "latest" Census) | 1 |  |
| McKiernan & Fleming (2017)[22] | Canada (Ontario, Saskatchewan, Alberta, Nova Scotia) | 77 | Youth. | [14-19] | 47:53 | Focus groups. | “A majority of participants recognized that cannabis can affect driving” and were able to cite reasons why (e.g., slowing down reaction time), but felt that these effects were dependent on factors such as how much he or she smoked and individual tolerance. | 0.95 |  |
| Fischer et al (2014)[23] | Canada (Ontario) | 248 | University students who drove within 4 h of cannabis use within the past 12 months. | 21.4 ± 2.6 | 71:29 | Telephone survey. | High- v.s., low-frequency DACU was associated with perception of one’s own ability to drive not being impaired by cannabis use (R²=.08) and expectation to DACU in the next 12 months (R²=.18, p's<.0001). | 0.88 |  |
| Jonah (2013)[24] | Canada | 1,500 | General population; licensed drivers who drove within the past 30 days. | NR | NR | Telephone Interview and online survey. | Over half of respondents were very concerned about DACU (58%) and strongly agreed that cannabis impairs driving (68%). *Weighted sample (region, age, sex of licensed drivers in Canada) | 0.83 |  |
| Macdonald et al (2008)[25] | Canada (Ontario) | 467 | Drug users in treatment program. | [18+] | NR | In-person survey. | Almost one third (28%) indicated that they “drive normally when under the influence of cannabis”. One fifth (20%) indicated that cannabis their reduced driving ability and a few (8%) indicated they drive better after cannabis use. | 0.79 |  |
| Porath-Waller (2008)[26] | Canada (Ontario) | 793 | Grade 9-12 high school students. | 15.9 ± 1.37 | 40:60 | In-person survey administered in schools. | Reported driving ability, dangerousness of driving, likelihood of having an accident, and peer acceptability of driving 1 - 12 h after using cannabis predicted intention to DACU within 1 h of using cannabis (R²=.36 - .47, R²=.36 - -.44, R²=-.26 - -.31, and R²=.34 - .48, respectively, p's < .0001). Those who used cannabis regularly reported greater intention to DACU and perceived little risk associated with DACU than those who did not. | 1 |  |
| Fischer et al (2006)[27] | Canada (Ontario) | 45 | University students who drove within 4 h of cannabis use within the past 12 months. | [18 – 28]  80.0% <24 | 58:42 | Face-to-face interviews. | The majority of respondents (95%) believed that cannabis produced some impairment for driving; however, less than two thirds (58%) believed they were affected by any impairment themselves and most of these believed that they had ways to compensate for some of the effects. Most (62%) respondents reported a certain or very likely chance of DACU in the next year. High frequency cannabis use (i.e., multiple times a week or more) was associated with high frequency of past-year DACU (i.e., ≥ 6 episodes) and a high level of anticipated likelihood of DACU in the next year (p's<.01). | 0.8 |  |
| Terry & Wright (2005)[28] | England | 132 | General population; drivers who used cannabis at least monthly and university students who had smoked cannabis. | 25.7 ± 6.6 | 57:43 | Mailed surveys and face-to-face interviews. | Of the 50 regular cannabis users (RCUs) who reported DACU, few (12%) believed their driving to be very much impaired (58% slightly impaired, 6% not at all impaired, and 24% improved). Of the 16 students who reported DACU, few (13%) believed their driving to be very much impaired (69% slightly impaired, 13% not at all impaired, and 6% improved). Most (64%) respondents were not really bothered by DACU or thought it was not a problem, whereas the rest said they really shouldn’t do it, but that it was not as bad as driving after drinking alcohol. Cannabis users were very willing to DACU (often combined with alcohol). | 0.82 |  |
| Danton et al (2003)[29] | England | 29 | Youth. | 19  [16 - 25] | 76:24 | Focus groups. | DACU was generally considered to be acceptable, possibly due to beliefs that cannabis does not impair ability to drive. Young people were risk averse when it came to drinking and driving but willing to take risks with DACU. | 0.95 |  |
| Malhotra et al (2017)[30] | New Zealand | 434 | General population; drove at least once within the last 12 months. | 34.54 ± 14.62 [17 - 74] | 36:64 | Online survey. | Cannabis users who drove reported lower impairment ratings (2.69/5; 1 not at all impaired - 5 ‘very impaired’ ) than users who did not drive (3.93/5), who in turn reported lower impairment ratings than cannabis non-users (4.40/5). Most (57%) reported making the decision not to drive within 3 h of using cannabis, mostly (34%) due to the belief that ability to drive was negatively affected. | 0.88 |  |
| Hammond (2009)[31] | New Zealand | 1,166 | General population. | 38.1 ± 13.6  [15 - 86] | 44:56 | Telephone interviews and online survey. | Respondents who had DACU thought it was less risky than those who had never DACU but used cannabis (t=-10.7, p<.001). Most (59%) cannabis users said they were ‘very likely’ or ‘somewhat likely’ to DACU. Among drivers who had DACU, most (58%) said there was no change in their driving ability, some (21%) said it was better and some (22%) said it was worse. | 0.91 |  |
| Ortiz-Peregrina et al (2022)[32] | Spain | 64 | General population; alcohol and/or cannabis users. | 24.3 ± 4.5  [19 – 43] | 58:42 | Between-subject experiments. Participants drank 300 ml and 450 ml of red wine or smoked cannabis ad libitum and drove a simulator. | Most (68%) cannabis users reported that their driving is slightly worse under the influence of cannabis; a fifth (19%) reported that their driving is much worse. Few (13%) reported that they feel no change or an improvement in their driving. | 0.96 |  |
| Pino et al (2016)[33] | Spain | 3,819 | Young adults. | 21.6 ± 3.63 [18–29] | 47:53 | In-person survey. | Two in five (42%) hashish users thought that DACU does not cause problems in driving, compared with one in ten (9%) non-users. Higher levels of consumption predicted lower accident risk perception (F=85,331, p<.0001). | 0.96 |  |
| Albery et al (2000)[34] | United Kingdom | 71 | Out-of-treatment illicit drug users who drove within the past year. | NR | NR | In-person interviews. | Compared to those who frequently used illicit drugs and drove, subjects who never or sometimes used illicit drugs and drove agreed more that using cannabis decreases driving skills and that the chances of having an accident are greater after taking cannabis (1.78; 1.78, respectively, v.s., 2.31 and 2.54; 2.31 and 2.50, respectively, where 1 - strongly disagree and 5 - strongly agree). Subjects who never used illicit drugs and drove agreed that heroin, methadone, and alcohol were less likely to lead to accidents, and impaired driving skills more than cannabis. Subjects who sometimes or frequently used illicit drugs and drove agreed that alcohol was less likely to lead to accidents and would impair driving skills more than cannabis, stimulants, heroin, and methadone (p’s<.05). | 1 |  |
| Miller et al (2024)[35] | United States | 30 | General population; drivers who used cannabis at least monthly. | Median: 32.5 [21 – 63] | 77:23 | Driver simulator study; participants were dosed with cannabis (6.18% THC) then drove at 30, 90, and 180-minutes post-dose. | Most (21 of 30) participants agreed with the statement that they could drive safely after using cannabis (14 somewhat agree, 7 strongly agree), and among those who agreed, 4 believed they were a better driver after consuming cannabis. | 0.96 |  |
| LoParco et al (2024)[36] | United States & Israel | 2,222 | General population. | Survey: 32.19 ± 7.74 [18–45]  Interviews: US = 36.5 ± 6.3, Israel = 29.35 ± 6.2 | Survey: 50:50  Interviews:  US = 57:43,  Israel = 58:42 | Online survey and online interviews. | The average perceived DACU vs. alcohol MVC risk was 2.90 (SD=1.29; 1 - cannabis is much less risky than alcohol to 5 - cannabis is much riskier than alcohol. In the interviews, some participants mentioned that MVC risk depends on the amount consumed, tolerance/previous experience, and potency. Several participants mentioned that detecting DACU was more difficult for law enforcement than detecting driving after alcohol use. No significant association among recreational cannabis legalization and DACU risk perception among US participants. | 0.96 (quantitative); 0.75 (qualitative) |  |
| Auguste & Zambrano (2023)[37] | United States (Connecticut) | 424 | General population; cannabis users. | [18+] | 45:48 | Online survey. | Higher frequency of cannabis use predicted lower self-reported impact of both recreational and medical cannabis on driving ability (t(218)=-7.787, p<.001 and t(173)=3.589, respectively, p’s<.001). | 0.92 |  |
| Eichelberger (2023)[38] | United States (Connecticut, Delaware, New York, North Carolina, South Carolina, Virginia) | 3,045 | General population; drivers who drove at least once within the past month. | [18+] | 47:53 | Telephone interviews. | About a third (29-41% across states) believed cannabis use and driving was a problem in their community. About two thirds (63-73% across states) agreed DACU increases crash risk. Drivers who reported using cannabis in the past year were significantly less likely to agree that it increases crash risk (43%) compared with those who did not.  *Weighted sample (age group within each state based on 2020 U.S. Census). | 1 |  |
| Hultgren et al (2023)[39] | United States (Washington) | 1,941 | Young adults. | 21.78 ± 2.30  [18-25] | 32:68 | Online survey. | The majority (85%) thought driving after drinking alcohol was ‘Totally Unacceptable’ (v.s. ‘Somewhat Unacceptable’, ‘Somewhat Acceptable’, and ‘Totally Acceptable’) whereas one-third (34%) reported DACU as ‘Totally Unacceptable’. Compared to those who thought DACU was ‘Totally Unacceptable’, those who endorsed any other response option had 11.90 times greater odds to DACU (within the past 30 days).  *Weighted sample (sex assigned at birth, geographic region, and race/ethnicity based on 2010 US Census data) | 0.96 |  |
| Brown et al (2022)[40] | United States (Colorado, Iowa, Illinois) | 3,045 | General population; history  of cannabis use. | [25-40] | 59:41 | Online survey. | Older age at first use, being female, and residing in CO (where recreational cannabis is legal) was associated with decreased perception of being able to safely drive within 2 h of cannabis use (p’s<.0001). | 0.96 |  |
| Benedetti et al (2021)[41] | United States | 11,816 | General population. | [19+] | 49:51 | Online survey (Traffic Safety Culture Index 2013–2017 Survey). | One in ten (10%) reported that DACU was acceptable. Male, younger, lower-income, and lower-education respondents more often reported DACU to be personally acceptable (p's < .001).  *Weighted sample (selection probability and non-response; age, gender, race/ethnicity, education, census region, urbanicity, household composition, and household income based on Census Bureau’s Current Population Survey 2013– 2017) | 0.92 |  |
| Scott et al (2021)[42] | United States | 2,084 | General population. | [18+] | 47:53 | Mailed and online survey. | DACU (frequency and recency) was predicted by willingness to DACU, which in turn influenced intention to DACU. Willingness and intention were influenced by control beliefs, norms, and attitudes toward people who never DACU. Intention was also influenced by attitudes toward people who DACU. Intention had a stronger influence on DACU than willingness alone (p’s<.05). *Weighted samples (mailed survey sample weighted to match distribution of age based on U.S. census 2015; online sample was weighted to match the percentage of young adults based on U.S. census 2015) | 0.96 |  |
| Teeters et al (2021)[43] | United States | 77 | College student cannabis users with access to a vehicle who drove under the influence of cannabis at least 3 times within the past 3 months. | 21.49 ± 3.75 [18+] | 34:66 | Proof-of concept pilot study that examined the preliminary efficacy of a mobile phone–based intervention with personalized feedback and text messaging to increase perceptions of dangerousness of DACU. | At baseline, participant perceptions of dangerousness of DACU within 2 h of using cannabis was rated 1.55 (1 - not at all dangerous to 4 - very dangerous). Participants in a personalized feedback condition with interactive text messaging reported significantly greater increases over time in the perception of dangerousness of DACU relative to controls who received information about drug and alcohol use. | 0.96 |  |
| Borodovsky et al (2020)[44] | United States | 3,010 | General population; past-month cannabis users who ever drove under the influence of cannabis. | [18-65] | 56:44 | Online survey. | Higher intoxication levels perceived as safe for driving were associated with more frequent DACU (ρ=.46). For each unit increase in the reported intoxication level perceived as safe for driving, the odds of DACU in the past month increased from 18% to 68% (ORs: 1–9 days=1.18; 10–19 days=1.40; 20–30 days=1.68). | 1 |  |
| Earle et al (2020)[45] | United States | 311 | Undergraduate students in psychology courses. | 18.7 ± 1.02 | 34:66 | In-person survey administered in undergraduate psychology courses. | Respondents indicated that DACU was not acceptable (1.69; 1 - unacceptable to 7 to acceptable). Attitudes toward DACU, subjective norms, and perceived behavioral control predicted intentions to DACU (β=.36, .05, and .28 respectively, p<.001). | 0.92 |  |
| Lensch et al (2020)[46] | United States | 17,112 | General population. | Sale of recreational cannabis:  Legal; 39.8 ± 16.0  Illegal; 40.1 ± 14.5  [16-65] | Sale of recreational cannabis:  Legal; 51:49 Illegal; 50:50 | Online survey (International Cannabis Policy Study – Wave 1). | Those living in states with legal sale of recreational cannabis (LSRC) believed that driving high increases the risk of accident ‘a lot’ compared with those in states with no LSRC (57% vs. 52%, aPR=1.10).  *Weighted sample (age, sex, education, race, sexual identity based on Statistics Canada 2016-2017 and U.S. Census Bureau 2013-2018) | 1 |  |
| AAA Foundation for Traffic Safety (2019)[47] | United States | 3,349 | General population. | [16 - 75+] | NR | Telephone and paper survey. | Seven in ten (70%) of respondents considered DACU within 1 h of using cannabis to be very or extremely dangerous. *Weighted sample (probability of selection based on gender, age, race, education, region, metro-status, number of people aged 16+ in household, and household income as per 2016 U.S. Census data and nonresponse) | 0.95 |  |
| Duckworth & Lee (2019)[48] | United States (Washington) | 511 | Young adults participating in a separate longitudinal study on who drank alcohol at within the past year. | 22.20 ± 1.74 | 42:58 | Online survey. | Participants believed it was unsafe to DACU (M=0.48, where 0 - rarely safe, 3 - always safe). Relative to single substance users, simultaneous cannabis and alcohol (SAM) use was associated with thinking that DACU was safer (β=.56, p<.0001). SAM users were more likely to think that DACU was safer than cannabis and alcohol co-users (β=.37, p<.001). | 1 |  |
| Eichelberger (2019)[49] | United States (Washington) | 2,355 | General population; licensed drivers. | [16+] | 53:47 | Roadside survey. | “The odds of drivers being THC-positive were significantly lower among those who perceived that ability to drive safely within 2 h of cannabis use very likely (OR=.60; p<.01) relative to other drivers.” “THC-positive drivers’ perceptions that cannabis is very likely to impair driving decreased significantly from 45% before retail sales to 17% 1 year after”; THC-negative drivers' perceptions did not change (52% before retail sales and 56% 1 year after).” “The odds of perceiving cannabis impairment as very likely did not differ significantly 6 months (OR=1.02; p=.88) or 1 year (OR=1.09; p=.49) after legalization.” *Weighted sample (relative size of the driving population by region based on Washington State Government’s Office of Financial Management 2010) | 0.92 |  |
| Berg et al (2018)[50] | United States | 649 | Young adults who used cannabis within the past 30 days. | 24.48 ± 5.10 [18–34] | 56:44 | Online survey. | The majority (62%) agreed or strongly agreed that they felt completely in control of their driving after using cannabis (1 - completely to 5 - agree completely). Less concern about DACU was associated with DACU (at least once in the past 30 days). | 0.91 |  |
| Cavazos-Rehg et al (2018)[51] | United States | 193 | General population; used cannabis extracts within the past 30 days (study 1) or within past 6 months (study 2). | Median: 24 (study 1); 25 (study 2)  [18+] | 73:27 | Telephone and online survey. | Two in five (39%) participants considered driving after using cannabis extracts to be a risky behavior (24% believed it to be safe and 32% believed that the level of risk depended on one’s response to cannabis, tolerance level, and/or amount/type of cannabis used). Over a third (37%) reported that they would feel comfortable DACU immediately after using extracts and a third (35%) believed that they need to wait at least 1 h. Participants who perceived DACU as safe or who felt that DACU safety was determined by such factors as one’s tolerance or type of cannabis used were more likely to DACU (51% and 54%, respectively; X^2^=33.3, p<.001). | 0.9 |  |
| Cuttler, Sexton & Mischley (2018)[52] | United States | 1,773 | General population; used cannabis within the past 90 days. | 34.23 ± 13.18  [16+] | 54:46 | Online survey. | Half (52%) indicated that cannabis does not impair driving ability. A third (30%) of those who reported believing that cannabis impairs driving ability also reported that they drive within 1 h of using cannabis. No significant differences were found between exclusively medical, exclusively recreational, and mixed cannabis users or in Washington respondents surveyed before- and after-legal sales. “Older age, less education, higher frequency of cannabis use, higher quantity of cannabis use, and driving within 1 h of use were significant predictors of beliefs that DACU is safe” (p's<.001). | 0.92 |  |
| Greene (2018)[53] | United States (Montana) | 72 | Young adults residing in rural areas. | 20.2 ± 2.1  [18 - 25] | 49:51 | Focus groups. | Participants believed that cannabis had the potential to impair or improve driving ability; the consensus was that DACU is benign with respect to crash risk, although some mentioned that using cannabis could result in minor impairment. Participants expressed that the degree to which cannabis impaired or improved driving ability depended on individual characteristics (e.g., tolerance, compensatory behaviours) and characteristics of the occasion and cannabis (e.g., type and amount used, concurrent use of other drugs). | 0.9 |  |
| Wadsworth & Hammond (2018)[54] | United States | 4,079 | Youth. | 17.59 ± 1.18 [16–19] | 53:47 | Online survey (International Tobacco Control Policy Evaluation Project Tobacco and Youth E-cigarette Survey). | “Almost half of youth in Prohibited, New and Established non-medical cannabis markets reported that driving a car within 2 h of using cannabis increases the risk of an accident by ‘a lot’ (44%, 44% and 42%, respectively).” Findings were inconclusive between youth in jurisdictions with prohibited, new and established non-medical cannabis markets. *Weighted sample (smoking frequency; geographic region and language in Canada; sex by smoking, age by smoking and age by race/ethnicity in the United states; sample size within each country/regulatory condition)  *Sample may partially overlap with Wadsworth & Hammond 2019 | 0.95 |  |
| Ward et al (2018)[55] | United States | 941 | General population. | [18+] | 49:51 | Panel (AmeriSpeak) distributed survey. | Cultural components (attitudes and norms) reliably predicted past DACU behavior, general DACU willingness, and future DACU intention. Intention and willingness both fully moderate the relationship between cultural components and DACU. Specifically, the probability of reporting past DACU behavior, willingness, and intention to DACU was 1.59, 1.40, and 1.71 times greater, respectively, among respondents with positive attitudes towards DACU; the probability of reporting willingness and intention to DACU was 1.60 and 1.74 times greater among respondents believing that DACU was normal among cannabis users (p's<.0001). | 1 |  |
| Arterberry et al (2017)[56] | United States | 897 | University students. | 19.01 ± 1.88 | 45:65 | Online survey. | Respondents who perceived DACU to be more dangerous were less likely to DACU (r=-.51, p<.001). | 0.91 |  |
| McGinty et al (2017)[57] | United States | 979 | General population. | [21+] | 45:55 | Online survey. | Half (52%) of respondents agreed with public anti-legalization arguments asserting that the policy would increase DACU-related MVCs. Residents of states where recreational cannabis was legal were less likely than those in states where cannabis was illegal to endorse anti-legalization arguments related to potential of increased MVCs due to legalization (46% vs. 55%, p<.01). | 1 |  |
| Ward et al (2017)[58] | United States (Washington) | 416 | General population; licensed drivers. | [16+] | 58:42 | Roadside survey. | Most (84%) respondents agreed that cannabis impairs driving ability. One in ten (12%) agreed that DACU is enjoyable. One in ten (11%) reported future intention to DACU. Drivers who reported believing cannabis does impair driving ability were 2.6 times more likely to not report intention to DACU in the next 6 months (p<.0001). | 1 |  |
| Allen et al (2016)[59] | United States (Washington & Colorado) | 865 | General population; used cannabis within the past 30 days. | [18+] | 40:60 | Online survey. | One third (33%) agreed or strongly agreed (1 - strongly disagree to 5 - strongly agree) that they could safely DACU. Subjects who reported being high at the time of survey administration (16%) were more likely to agree that they could DACU safely (OR=3.13, p<.001), that it is okay to drive a little bit high (OR=2.74, p<.001) or that it is acceptable to DACU in certain situations (OR=2.17, p<.001). Frequent users (21–30 days per month) and heavier users (high and medium usage tertiles) were more likely to agree that they could DACU safely (OR=2.66 and 2.30, respectively, p's<.000) and that it is okay to drive a little high (OR=2.15 and 2.87, respectively, p's<.000).  *Sample used in Davis et al. 2016 | 0.96 |  |
| Davis et al (2016)[60] | United States (Washington & Colorado) | 865 | General population, used cannabis within the past 30 days. | [18+] | 40:60 | Online survey. | Respondents disagreed slightly that they could safely DACU (3.29; 1 – strongly agree to 5 – strongly disagree), that if they are just a little bit high, they don't think their ability to drive is impaired (3.24), that being high on cannabis or hashish doesn't affect their driving (3.51), and that driving high is not a big deal (3.27). Greater perception that it is unsafe to DACU was associated with a lower past-month incidence of driving ≥5 times within 1 h of using cannabis or hashish (OR=0.26, p<0.01), lower odds of any DACU in the past year (OR=0.31, p<.01), and lower odds of agreeing or strongly agreeing that they might DACU even though they know they should not (OR=0.25, p<0.01).  *Sample used in Allen et al. 2016 | 1 |  |
| Aston et al (2016)[61] | United States | 151 | Non–treatment-seeking frequent cannabis users (used cannabis at least once per week within the past month, at least 10 times within the past 6 months). | 21.54 ± 3.16 [18–30] | 64:36 | Survey (online or in-person unclear). | Respondents reported it was slightly dangerous (1.98; 1 - not at all dangerous to 4 - very dangerous) to DACU. Smoking cannabis while driving was negatively correlated with perceived dangerousness, negative expectancies, perceived peer norms, and perceived negative consequences of DACU (p's<.05). Less salient perceived driving-related peer norms and perceptions that DACU is less dangerous were associated with increased frequency of DACU. | 0.91 |  |
| Eichelberger (2016)[62] | United States | 4,525 | General population; drivers. | [18+] | 50:50 | Telephone interviews. | Three fourths (78%) of drivers agreed that DACU increases the likelihood of a crash. Most drivers (75%) who had DACU did not think that cannabis affected their driving. Drivers in states with legal recreational cannabis more often said that DACU is a problem relative to those in states without legalized recreational cannabis (43% vs. 28%). Drivers who supported legalized recreational cannabis were significantly less likely to believe that DACU increases crash risk compared with those who opposed it (61% vs. 91%).  *Weighted sample (age, sex, and population by state based on United States Census Bureau 2015 data) | 0.95 |  |
| Otto et al (2016)[63] | United States | 2,053 | General population (survey 3: past-30-day cannabis users). | [21+], *n* = 820; [18 - 30], n = 1,233 | 47:53 | Mailed and online survey. | Cannabis users (compared with non-users) and users who reported not driving within 4 h of using cannabis in the past 12 months (compared with those who had) had more positive attitudes towards DACU, greater intention to DACU, greater willingness to DACU in a variety of circumstances, held normative beliefs (both injunctive and descriptive) that are more supportive of DACU, and had greater perceived control over DACU in certain situations (p's<.000). Negative attitudes towards DACU predicted lower willingness to DACU among cannabis users (p's<.001). There were no differences in values, beliefs, or attitudes between states with and without legalized use of recreational or medical cannabis. *Weighted sample (gender, age based on general population estimates) | 1 |  |
| Kohn et al (2014)[64] | United States | 444 | University students. | 19.4 ± 1.3 | 51:49 | In-person survey administered in university classes. | A minority of students (18%) disagreed that cannabis impairs driving. | 0.88 |  |
| Arterberry et al (2013)[65] | United States | 506 | College students who ever used cannabis who had access to a car or drove at least once per month. | 18.3 ± 1.1 | 51:49 | In-person survey administered in college classes. | Respondents expressed that it was dangerous to drive within 2 h after smoking cannabis (2.30; 1 - not at all dangerous to 4 - very dangerous). Greater peer acceptance and greater perceptions of danger were associated with lower levels of DACU and riding with an impaired driver (RWHD) among cannabis smokers who engaged in these behaviors (r's=-.22 to -.40, p's<.001). After controlling for cannabis use, gender, and other cognitive predictors, greater perceived dangerousness of DACU was associated with decreased odds of engaging in DACU and RWHD (OR=.55, p<.002 and OR=0.69, p<.022, respectively). | 1 |  |
| Ginsburg et al (2008)[66] | United States | 5,665 | Grade 9 - 11 students. | NR | 51:49 | In-person survey administered in high schools. | Most students (72%) indicated that smoking cannabis would make "a lot of difference" in driving safety. DACU was ranked 3rd on driving safety risk (after alcohol and driving and texting and racing while driving).  *Weighted sample (gender, age, and race/ethnicity, oversampling and differing response rates among schools) | 1 |  |
| McCarthy et al (2007)[67] | United States (Missouri) | 599 | University students. | 18.54 ± 0.86 | 41:59 | In-person survey. | Perceptions that DACU is less dangerous were associated with past-month and past-year cannabis use frequency (r=.47 and r=.52, respectively, p's<.01). Greater perceived dangerousness of DACU was related to greater frequency of DACU (OR=1.79, p<.01). | 1 |  |
| Townsend et al (1998)[68] | United States | 11,847 | General population; drove a motor vehicle at least once within the 12 months. | [16+] | 50:50 | In-home personal interviews (1996 National Household Survey on Drug Abuse survey). | Over half (56%) reported that cannabis use did not affect their ability to drive safely at all.  *Weighted sample (selection probabilities of respondents) | 0.78 |  |
| Wechsler et al (1984)[69] | United States (Massachusetts) | 623 | Grade 7 and 10 students. | [16+] | 55:45 | In-person survey administered in schools. | Few respondents (15%) reported that cannabis did not affect driving ability. Heavier drinkers, cannabis users, and other drug users were more likely to believe that cannabis does not affect driving ability (25%, 22%, and 25% vs 9-14%, respectively). | 0.80 |  |
| Grilly (1981)[70] | United States (Ohio) | 400 | University students. | NR | NR | In-person survey administered in university. | Cannabis was perceived by the majority of respondents to be detrimental to driving skills (both self; 68-73%, and others; 86-90% across years 1975, 1977, and 1980). Respondents demonstrated comparative optimism (i.e., they were more likely to believe that their own driving skills were less impaired by cannabis than other's). | 0.83 |  |

**NR = not reported; DACU = driving under the influence of cannabis; OR = odds ratio; aPR = adjusted prevalence ratio*

**References**

1. Arkell, T.R., et al., *Driving-related behaviors, attitudes, and perceptions among Australian medical cannabis users: results from the CAMS 20 survey.* J Cannabis Res, 2023. **5**(1): p. 35.

2. Mills, L. and J. Freeman, *Investigating predictors of driving immediately after consuming cannabis: a study of medical and recreational cannabis users in Australia.* Transp. Res. F: Traffic Psychol. Behav., 2023. **96**: p. 213-21.

3. Mills, L., J. Freeman, and B. Rowland, *Australian daily cannabis users' use of police avoidance strategies and compensatory behaviours to manage the risks of drug driving.* Drug Alcohol Rev, 2023. **42**(6): p. 1577-86.

4. Arkell, T.R., et al., *Driving-related behaviours, attitudes and perceptions among Australian medical cannabis users: results from the CAMS 18-19 survey.* Accid Anal Prev., 2020. **148**: p. 105784.

5. Matthews, A.J., et al., *Driving under the influence among frequent ecstasy consumers in Australia: trends over time and the role of risk perceptions.* Drug Alcohol Depend, 2014. **144**: p. 218-224.

6. Barrie, L.R., S.C. Jones, and E. Wiese, *“At least I’m not drink-driving”: Formative research for a social marketing campaign to reduce drug-driving among young drivers.* Australasian marketing journal, 2011. **19**(1): p. 71-75.

7. Swift, W., C. Jones, and N. Donnelly, *Cannabis use while driving: a descriptive study of Australian cannabis users.* Drugs: Educ Prev Policy, 2010. **17**(5): p. 573-586.

8. Adams, K., L. Smith, and N. Hind, *Drug driving among police detainees in Australia.* Trends Issues Crime Crim Justice, 2008(357): p. 1-6.

9. Jones, C., et al., *Preventing cannabis users from driving under the influence of cannabis.* Accid Anal Prev, 2006. **38**(5): p. 854-61.

10. Lenné, M.G., et al., *Attitudes and experiences of people who use cannabis and drive: implications for drugs and driving legislation in Victoria, Australia.* Drugs: Educ Prev Policy, 2001. **8**(4): p. 307-13.

11. Aitken, C., M. Kerger, and N. Crofts, *Drivers who use illicit drugs: behaviour and perceived risks.* Drugs: Educ Prev Policy, 2000. **7**(1): p. 39-50.

12. Wickens, C.M., et al., *Risk perceptions of driving under the influence of cannabis: comparing medical and non-medical cannabis users.* Transp. Res. F: Traffic Psychol. Behav., 2023. **95**: p. 36-45.

13. Huỳnh, C., et al., *Typologies of Canadian young adults who drive after cannabis use: a two‐step cluster analysis.* Behav Sci Law, 2022. **40**(2): p. 310-30.

14. Colonna, R., et al., *Exploring youths’ beliefs towards cannabis and driving: a mixed method study.* Transp Res Part F Traffic Psychol Behav, 2021. **82**: p. 429-39.

15. McDonald, A.J., et al., *Driving under the influence of cannabis risk perceptions and behaviour: a population-based study in Ontario, Canada.* Prev Med, 2021. **153**: p. 106793.

16. Goodman, E.S., L.-T. Cesar, and H. David, *Risk perceptions of cannabis vs. alcohol-impaired driving among Canadian young people.* Drugs: Educ Prev Policy, 2020. **27**(3): p. 205-212.

17. Woods-Fry, H., R.D. Robertson, and W.G.M. Vanlaar, *Road Safety Monitor 2020: trends in marijuana use among Canadian drivers*. 2020, Traffic Injury Research Foundation: Ottawa (ON). p. 5 p.

18. Wadsworth, E. and D. Hammond, *International differences in patterns of cannabis use among youth: prevalence, perceptions of harm, and driving under the influence in Canada, England & United States.* Addict Behav, 2019. **90**: p. 171-175.

19. Wickens, C.M., et al., *Exploring perceptions among people who drive after cannabis use: collision risk, comparative optimism and normative influence.* Drug Alcohol Rev, 2019. **38**(4): p. 443-51.

20. Corporate Research Associates, *2018 Edmonton and Area Traffic Safety Culture Survey*. 2019, City of Edmonton, Traffic Safety Section: Edmonton (AB). p. 94 p.

21. EKOS Research Associates Inc, *Public opinion research on drug mpaired driving: baseline survey findings report*. 2017: Ottawa (CA).

22. McKiernan, A. and K. Fleming, *Canadian youth perceptions on cannabis*. 2017, Canadian Centre on Substance Abuse: Ottawa (ON).

23. Fischer, B., et al., *Factors associated with high-frequency cannabis use and driving among a multi-site sample of university students in Ontario.* Can J Criminol Crim Justice, 2014. **56**(2): p. 185-200.

24. Jonah, B., *CCMTA public opinion survey of drugs and driving in Canada*. 2013, Canadian Council of Motor Transport Administrators: Ottawa (ON).

25. MacDonald, S., et al., *Driving behavior under the influence of cannabis or cocaine.* Traffic Inj Prev, 2008. **9**(3): p. 190-194.

26. Porath-Waller, A.J., *Driving high: a study of student driving behaviours and attitudes towards marijuana use and driving [thesis]*. 2008, Ottawa (ON): Carelton University; 2008.

27. Fischer, B., et al., *Toking and driving: characteristics of Canadian university students who drive after cannabis use-an exploratory pilot study.* Drugs: Educ Prev Policy, 2006. **13**(2): p. 179-87.

28. Terry, P. and K.A. Wright, *Self-reported driving behaviour and attitudes towards driving under the influence of cannabis among three different user groups in England.* Addict Behav, 2005. **30**(3): p. 619-26.

29. Danton, K., et al., *Attitudes of young people toward driving after smoking cannabis or after drinking alcohol.* Health Educ J, 2003. **62**(1): p. 50-60.

30. Malhotra, N., N.J. Starkey, and S.G. Charlton, *Driving under the influence of drugs: perceptions and attitudes of New Zealand drivers.* Accid Anal Prev, 2017. **106**: p. 44-52.

31. Hammond, K., *Drug driving in New Zealand: a survey of community attitudes, experience and understanding*. 2009, New Zealand Drug Foundation: NZ. p. 99 p.

32. Ortiz-Peregrina, S., et al., *Comparison of the effects of alcohol and cannabis on visual function and driving performance. Does the visual impairment affect driving?* Drug and alcohol dependence, 2022. **237**: p. 109538-109538.

33. Pino, M.J., et al., *Legal and illegal substance consumption and traffic accident risk perception among Spanish young people.* Soc Indic Res, 2016. **129**(2): p. 835-845.

34. Albery, I.P., et al., *Illicit drugs and driving: prevalence, beliefs and accident involvement among a cohort of current out-of-treatment drug users.* Drug Alcohol Depend, 2000. **58**(1-2): p. 197-204.

35. Miller, R., et al., *Predicting changes in driving performance in individuals who use cannabis following acute use based on self-reported readiness to drive.* Accident analysis and prevention, 2024. **195**: p. 107376-107376.

36. LoParco, C.R., et al., *Driving under the influence of cannabis versus alcohol: a mixed-methods study examining perceptions and related risk behaviors among US and Israeli adults.* Addict. Behav., 2024. **148**: p. 107843.

37. Auguste, M.E. and V.C. Zambrano, *Self-reported impacts of recreational and medicinal cannabis use on driving ability and amount of wait time before driving.* Traffic Inj. Prev., 2023. **24**(3): p. 237-241.

38. Eichelberger, A., *Prevalence of alcohol, cannabis, and simultaneous use among drivers in six States.* Transp. Res. Rec., 2023. **2677**(11): p. 237-44.

39. Hultgren, B.A., et al., *Injunctive Norms and Driving Under the Influence and Riding With an Impaired Driver Among Young Adults in Washington State.* Journal of Adolescent Health, 2023. **73**(5): p. 852-858.

40. Brown, T., et al., *A study of self-reported personal cannabis use and state legal status and associations with engagement in and perceptions of cannabis-impaired driving.* Traffic Inj. Prev., 2022. **23**(sup1): p. S183-6.

41. Benedetti, M.H., et al., *Demographic and policy-based differences in behaviors and attitudes towards driving after marijuana use: an analysis of the 2013–2017 Traffic Safety Culture Index.* BMC Res Notes, 2021. **14**(1): p. 1-226.

42. Scott, B., et al., *Modeling the system of beliefs that influence driving under the influence of cannabis (DUIC) in Washington State.* Accid Anal Prev, 2021. **151**: p. 105988.

43. Teeters, J.B., S.A. King, and S.M. Hubbard, *A mobile phone-based brief intervention with personalized feedback and interactive text messaging is associated with changes in driving after cannabis use cognitions in a proof-of-concept pilot trial.* Exp Clin Psychopharmacol, 2021. **29**(2): p. 203-9.

44. Borodovsky, J.T., et al., *Perceived safety of cannabis intoxication predicts frequency of driving while intoxicated.* Prev Med, 2020. **131**: p. 105956.

45. Earle, A.M., et al., *Examining interactions within the theory of planned behavior in the prediction of intentions to engage in cannabis-related driving behaviors.* Journal of American College Health, 2020. **68**(4): p. 374-380.

46. Lensch, T., et al., *Cannabis use and driving under the influence: behaviors and attitudes by state-level legal sale of recreational cannabis.* Prev Med, 2020. **141**: p. 106320.

47. AAA Foundation for Traffic Safety, *2018 Traffic Safety Culture Index*. 2019, AAA Foundation for Traffic Safety: Washington (DC). p. 35 p.

48. Duckworth, J.C. and C.M. Lee, *Associations among simultaneous and co-occurring use of alcohol and marijuana, risky driving, and perceived risk.* Addict Behav, 2019. **96**: p. 39-42.

49. Eichelberger, A.H., *Marijuana use and driving in Washington State: risk perceptions and behaviors before and after implementation of retail sales.* Traffic Inj Prev, 2019. **20**(1): p. 23-29.

50. Berg, C.J., et al., *Marijuana use and driving under the influence among young adults: a socioecological perspective on risk factors.* Substance use & misuse, 2018. **53**(3): p. 370-380.

51. Cavazos-Rehg, P.A., et al., *Operating a motor vehicle after marijuana use: perspectives from people who use high-potency marijuana.* Subst Abus, 2018. **39**(1): p. 21-26.

52. Cuttler, C., M. Sexton, and L.K. Mischley, *Driving under the influence of cannabis: an examination of driving beliefs and practices of medical and recreational cannabis users across the United States.* Cannabis, 2018. **1**(2): p. 1-14.

53. Greene, K.M., *Perceptions of driving after marijuana use compared to alcohol use among rural American young adults.* Drug Alcohol Rev, 2018. **37**(5): p. 637-644.

54. Wadsworth, E. and D. Hammond, *Differences in patterns of cannabis use among youth: prevalence, perceptions of harm and driving under the influence in the USA where non‐medical cannabis markets have been established, proposed and prohibited.* Drug Alcohol Rev, 2018. **37**(7): p. 903-911.

55. Ward, N.J., et al., *Developing a theoretical foundation to change road user behavior and improve traffic safety: driving under the influence of cannabis (DUIC).* Traffic Inj Prev, 2018. **19**(4): p. 358-363.

56. Arterberry, B.J., H. Treloar, and D.M. McCarthy, *Empirical profiles of alcohol and marijuana use, drugged driving, and risk perceptions.* J Stud Alcohol Drugs, 2017. **78**(6): p. 889-898.

57. McGinty, E.E., et al., *Public perceptions of arguments supporting and opposing recreational marijuana legalization.* Prev Med, 2017. **99**: p. 80-6.

58. Ward, N.J., et al., *Cultural predictors of future intention to drive under the influence of cannabis (DUIC).* Transp Res Part F Traffic Psychol Behav, 2017. **49**: p. 215-225.

59. Allen, J.A., et al., *Association between self-reports of being high and perceptions about the safety of drugged and drunk driving.* Health Education Research, 2016. **31**(4): p. 535-541.

60. Davis, K.C., et al., *Correlates of marijuana drugged driving and openness to driving while high: evidence from Colorado and Washington.* PloS One, 2016. **11**(1): p. e0146853.

61. Aston, E.R., et al., *Risk factors for driving after and during marijuana use.* J Stud Alcohol Drugs, 2016. **77**(2): p. 309-316.

62. Eichelberger, A.H., *Survey of U.S. drivers about marijuana, alcohol, and driving*. 2016, Insurance Institute for Highway Safety: Arlington (VA). p. 1-17.

63. Otto, J., K. Finley, and N.J. Ward, *An assessment of traffic safety culture related to driving after cannabis use*. 2016, Center for Health and Safety Culture, Western Transportation Institute, Montana State University: Helena (MT). p. 79 p. Report No.:FHWA/MT-16-010/8882-309-02.

64. Kohn, C., et al., *Correlates of drug use and driving among undergraduate college students.* Traffic Inj Prev, 2014. **15**(2): p. 119-124.

65. Arterberry, B.J., et al., *Marijuana use, driving, and related cognitions.* Psychol of Addict Behav, 2013. **27**(3): p. 854-860.

66. Ginsburg, K.R., et al., *National young-driver survey: teen perspective and experience with factors that affect driving safety.* Pediatrics, 2008. **121**(5): p. e1391-403.

67. McCarthy, D.M., A.M. Lynch, and S.L. Pederson, *Driving after use of alcohol and marijuana in college students.* Psychol Addict Behav, 2007. **21**(3): p. 425-430.

68. Townsend, T.N., et al., *Driving after drug or alcohol use: findings from the 1996 National Household Survey on Drug Abuse*. 1998, Dept. of Health and Human Services, Substance Abuse and Mental Health Services Administration: Washington (DC). p. 119 p.

69. Wechsler, H., et al., *Alcohol and other drug use and automobile safety: a survey of Boston-area teen-agers.* J Sch Health, 1984. **54**(5): p. 201-3.

70. Grilly, D.M., *People's views on marijuana, other drugs & driving: an update.* J Psychoact Drugs, 1981. **13**(4): p. 377-379.
