# Supplementary material for: Attitudes toward driving after cannabis use: a systematic review
Source: J Cannabis Res. 2024 Sep 28;6:37. doi: 10.1186/s42238-024-00240-0 (PMC11439277; doi:10.1186/s42238-024-00240-0)
Supplement: Supplementary file 3 — Additional file 3. Thematic synthesis codes. [file 42238_2024_240_MOESM3_ESM.docx]

**Supplementary File 3 – Thematic Synthesis Codes**

**Codes**

1. Dangerousness/safety of DACU
2. Effects of cannabis on driving ability
3. Effects of cannabis accident risk
4. Perceived control to DACU OR while DACU
5. Willingness to DACU
6. Intention to DACU
7. Social perception of DACU/norms
8. Enjoyability of DACU
9. Acceptability of DACU
10. Morality of DACU
11. History of DACU
12. Past accident involvement while DACU
13. Past violations while DACU (e.g. receiving ticket)
14. Riding with impaired driver
15. Driving another while impaired
16. How easy it is to tell if a driver is under the influence of cannabis
17. Motives for DACU
18. Decided against DACU and why
19. Negative effects on driving ability (e.g. slower reaction time)
20. Positive effects on driving ability (e.g. more cautious driving)
21. Compensatory driving behaviours while under influence of cannabis
22. Time waited before driving
23. Associations between attitudes and actual DACU
24. Associations between attitudes toward DACU and intention to DACU
25. Associations between attitudes toward DACU and frequency of cannabis use
26. Sex/gender
27. Age
28. Education
29. Employment
30. Income
31. Ethnicity
32. Urbanicity/rurality
33. Driving experience (km driven, years driving, license type)
34. Cannabis use history (e.g. user, non-users, frequent user)
35. Amount of cannabis consumed
36. Mode of administration
37. Tolerance
38. Special populations (e.g. ecstasy users, medical cannabis users)
39. Beliefs about effect of cannabis on self vs others' driving
40. Comparisons: pre- v.s. post-legalization
41. Comparisons: jurisdictions where recreational cannabis is legal v.s. illegal
42. Beliefs about being apprehended by police OR penalties for DACU
43. Deterrence by penalties
44. Knowledge of DACU laws
45. Support of measures to prevent DACU
46. Education cannabis vs. alcohol
47. Comparisons: effects of cannabis v.s. alcohol v.s. cannabis and alcohol on driving
48. Comparisons: other drugs v.s. cannabis
